# Clove Oil–Loaded Zein Nanoparticles: Eugenol Release and Repellent Activity against *Sitophilus zeamais*

**DOI:** 10.1021/acsomega.5c09938

**Published:** 2026-02-03

**Authors:** Laurieth Góes de Jesus, Railan dos Santos Silva, Rosilene Aparecida de Oliveira, Carla Fernanda Favaro, Carlos Eduardo Pereira, Rodrigo Luis Silva Ribeiro Santos

**Affiliations:** † Departamento de Ciências Exatas, 74361 Universidade Estadual de Santa Cruz (UESC), Ilhéus 45662-900, Brasil; ‡ Centro de Formações Em Ciências Agroflorestais, 423878 Universidade Federal Do Sul da Bahia (UFSB), Itabuna 45600-923, Brasil

## Abstract

Pest and pathogen infestations represent major challenges in agriculture, compromising crop production and grain storage, with significant economic and social impacts on food security. Nanotechnology has emerged as a promising tool for pest management through the development of nanoparticles carrying bioactive compounds such as essential oils. Among these, clove essential oil extracted from *Syzygium aromaticum* stands out for its insecticidal and repellent activities, although its high volatility, susceptibility to photodegradation, and oxidation limit direct applications. Encapsulation in biodegradable polymeric matrices is an effective strategy to enhance stability and extend functionality. In this study, clove oil (CO) and its main component, eugenol (Eug), were incorporated into zein, a maize-derived protein, to investigate *in vitro* release kinetics and *in vivo* bioactivity against *Sitophilus zeamais*. CO was extracted from flower buds by hydrodistillation, yielding 10.3% (w/w) with 78.6% (v/v) Eug content. Zein nanoparticles (NPZ), along with CO- and Eug-loaded systems (NPZ-CO, NPZ-Eug), exhibited mean hydrodynamic diameters around 300 nm and spherical morphology. Storage assays indicated improved stability under refrigeration. *In vitro* release studies revealed gradual Eug release from NPZ-CO and NPZ-Eug over 200 min, best described by Korsmeyer–Peppas (R^2^ > 0.93) and Weibull (R^2^ > 0.94) models, indicating an initial burst followed by diffusion-controlled release. *In vivo* assays showed that, while the tested doses did not produce significant toxicity against *S. zeamais*, encapsulated formulations (NPZ-CO, NPZ-Eug) exhibited enhanced repellent activity compared to the free compounds after 24 h of exposure. Overall, these results indicate that zein-based nanocarriers can improve the stability of essential oils, leading to a more effective repellent response.

## Graphical abstract

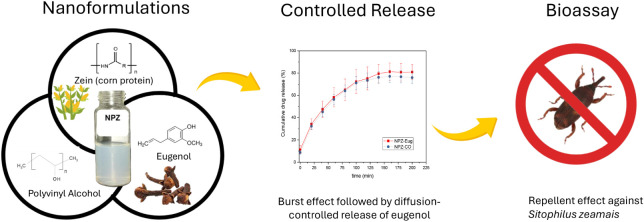

## 1 Introduction

Pest infestations and pathogenic agents represent one of the greatest challenges for farmers, compromising both crops and the storage of agricultural products. According to the Food and Agriculture Organization (FAO), up to 40% of global crops may be lost due to these factors, resulting in estimated damages of USD 220 billion.^1^ In the case of stored grains, the high nutrient availability makes them especially vulnerable to pests, contributing to global annual losses of up to 14%.^2^ Among the insects that attack stored maize, species from the orders Coleoptera and Lepidoptera stand out, with the maize weevil (*Sitophilus zeamais* of the family Curculionidae) being the most economically impactful pest, responsible for up to 13% of total postharvest losses worldwide.^3^ This pest is highly damaging due to its ability to cause cross-infestation, affecting grains both in the field and in warehouses, where it penetrates the stored mass. In addition, it has a high reproductive potential and a wide host range, including wheat, maize, rice, barley, and triticale. Its control is generally carried out through the application of synthetic insecticides such as neonicotinoids, carbamates, pyrethroids, and organophosphates.^4^ However, the excessive use of these compounds may harm the environment and human health.

Among the botanical alternatives under investigation, clove essential oil (CO), extracted from the flower buds of *Syzygium aromaticum*, stands out for its reported insecticidal, antimicrobial, and antiparasitic activity, mainly attributed to eugenol (Eug).^6^ However, its high volatility results in low persistence under field conditions, which limits its agricultural application. To overcome this limitation, nanotechnology techniques such as encapsulation have been employed. This process consists of incorporating the active substance within a polymeric matrix, aiming to promote its gradual release under specific conditions. In this way, the volatile constituents of the essential oil are protected from adverse environmental factors, resulting in greater environmental stability.^7^

Natural polymers have attracted particular interest for this purpose due to their biocompatibility and biodegradability.^8^ Among them, zein, a prolamin extracted from maize kernels, has emerged as a promising candidate because of its safety profile and regulatory acceptance for pharmaceutical and food applications. This protein exhibits excellent biocompatibility and biodegradability, making it an ideal material for the development of nanoformulations.^9,10^ Zein-based nanoparticles (NPZ) have already been used to encapsulate different essential oils and aromatic compounds, including clove oil, eugenol,^7^ geraniol, citronellal,^11^ limonene, carvacrol,^12^ and curcumin.^13^

Recent reviews have expanded the current understanding of the zein matrix as a nanoscale delivery material by examining both its structural behavior and its broad application potential. Several authors have described the intermolecular forces driving zein self-assembly, such as hydrophobic interactions, hydrogen bonding, electrostatic contributions, and van der Waals forces; and how these interactions govern particle formation, stability, and encapsulation efficiency.^30,66,67^ These works also outline the main preparation routes for obtaining zein-based nanocarriers, including antisolvent precipitation, pH-driven assembly, solvent evaporation, and chemical cross-linking, discussing the advantages and limitations of each method. Complementarily, another set of reviews emphasizes the functional use of zein nanostructures across food, pharmaceutical, agricultural, cosmetic, and environmental applications, highlighting their capacity to encapsulate hydrophobic bioactives, improve stability and bioaccessibility, and enable controlled-release performance.^40,68–70^

Despite these advances and the growing number of articles published summarizing the structural behavior and application potential of zein-based nanocarriers, no studies have reported the use of zein nanoparticles to deliver clove oil or eugenol specifically against *S. zeamais*. Moreover, although stabilizers such as pluronic,^11,54^ sodium caseinate,^33,53^ poloxamer,^71^ lecithin,^72^ and rhamnolipids^73^ are commonly employed in NPZ formulations, the use of poly­(vinyl alcohol) (PVA) as a stabilizing agent in the preparation of zein nanoparticles has not been previously described by other groups, representing a formulation innovation explored in the present work.

PVA is a water-soluble, nonionic synthetic polymer widely used to enhance the colloidal stability of nanoformulations, including polymeric nanoparticles,^15,74^ nanofibers^75^ and films.^76^ Its stabilizing effect arises mainly from steric hindrance, forming a protective hydrated layer around the particles that prevents agglomeration and can influence the interaction between the polymer matrix and encapsulated compounds.

Despite growing interest in essential oil-based nanoformulations, the relationship between nanoparticle stability, release kinetics, and biological efficacy remains poorly understood, particularly for zein nanoparticles stabilized with PVA and loaded with CO or Eug. Therefore, this study focuses on preparing these nanoformulations, characterizing their colloidal properties, evaluating the release kinetics of eugenol, and assessing their toxicity and repellent activity against *S. zeamais* under controlled laboratory conditions.

## 2 Materials and Methods

### 2.1 Chemicals and Reagents

Zein (20 kDa), poly­(vinyl alcohol) (PVA, 99% hydrolyzed, 89–98 kDa), and eugenol (99%) were purchased from Sigma-Aldrich. Dried flower buds of *Syzygium aromaticum* were obtained from local markets in the region of Valença, Bahia, Brazil (13° 22′ 50” S; 39° 4′ 56” W) in November 2022. All solvents used were of analytical grade.

### 2.2 Extraction and Analysis of Essential Oil

The essential oil was extracted from 60.2 g of dried clove buds in 1.3 L of distilled water using a Clevenger apparatus for 5 h. The oil was separated from the hydrolate by extraction with dichloromethane, dried over anhydrous sodium sulfate, and concentrated. The yield was expressed as a percentage based on the ratio between the mass of the obtained oil and the dry plant material used (% w/w), in triplicate. The extracted oil was stored at −10 °C until use. The chemical composition of the samples was analyzed by gas chromatography with flame ionization detection (GC-FID) and gas chromatography coupled to mass spectrometry (GC-MS). GC-FID analyses were performed using a GC-2010 Plus gas chromatograph (Shimadzu) equipped with a fused silica capillary column VF-5 ms (30 m × 0.25 mm i.d., 0.25 μm film thickness) coated with 5% phenyl–95% dimethylpolysiloxane. Helium was used as the carrier gas at a constant flow rate of 1.2 mL/min (10 psi). One microliter of the sample was injected in split mode (1:10), with injector and detector temperatures set to 250 °C. The oven temperature program was set from 130 to 240 °C at a rate of 3 °C/min.

The qualitative analysis was carried out on a GC-MS QP 2010 SE mass spectrometer (Shimadzu) equipped with a triple quadrupole analyzer. The column and temperature conditions were identical to those used in the GC-FID analysis. The instrument was operated in electron impact mode at 70 eV, with a scan rate of 1 scan per second over the mass range of 50–600 Da. The ion source temperature was set to 200 °C and the interface to 250 °C. Identification of the oil components was based on the analysis of fragmentation patterns in the mass spectra, confirmed by comparison with the spectra available in the instrument’s database (NIST 11), as well as by comparing their retention indices with those of known compounds, obtained through the injection of a standard mixture containing a homologous series of alkanes (C8–C26, Sigma–USA), and with data reported in the literature.^14^

The eugenol (Eug) content in CO was determined using UV–vis molecular absorption spectroscopy, with an external standard calibration curve previously prepared in 85% (v/v) ethanol over a working range of 15–57 μg mL^–1^ (y = 0.017x + 0.0265, R^2^ = 0.996). For this purpose, the CO sample was diluted 200-fold, and the electronic absorbance spectrum was recorded in the range of 200–600 nm.

### 2.3 Synthesis and Analyses of Zein Nanoparticles

#### 2.3.1 Preparation and Physicochemical Characterization

Zein nanoparticles (NPZ), as well as zein nanoparticles loaded with eugenol (NPZ-Eug) and clove oil (NPZ-CO), were prepared using the nanoprecipitation method, as described in our previous study.^8^ First, a 0.3% (w/v) zein suspension in 85% (v/v) ethanol was prepared and magnetically stirred at ∼500 rpm (IKA Werke RT 10 power) for approximately 8 h at room temperature. The solution was then heated for 15 min at 75 °C and subsequently filtered through Millex syringe filters (0.45 μm) to remove particulate residues. Next, 1 mL of this zein solution was mixed with 9 mL of a 0.6% (w/v) poly­(vinyl alcohol) (PVA) solution, previously prepared in distilled water and adjusted to pH 4.0 with HCl (1.0 M), resulting in the immediate formation of the nanoparticulate suspension (NPZ). The suspension was kept under vigorous magnetic stirring (∼800 rpm) for 2 h at room temperature, after which the final volume was adjusted to 10 mL with distilled water. For the preparation of zein nanoparticles loaded with eugenol (NPZ-Eug) and clove oil (NPZ-CO), the same procedure was followed, except for the addition of approximately 15 mg of Eug and CO, respectively. The obtained nanoparticles were characterized by dynamic light scattering (DLS) and electrophoretic light scattering (ELS) using a Zetasizer Nano-ZS Zen 3600 (Malvern Instruments, UK).

The encapsulation efficiency (%EE) was calculated from [Eug]_(found)_ and [oil]_(initial)_ (eq 1), where [oil]_(initial)_ refers to the concentration of the active ingredient added to the formulation (eugenol for NPZ-Eug and clove oil for NPZ-CO), and [Eug]_(found)_ corresponds to the eugenol concentration quantified in the sample by UV–vis spectroscopy (λ = 280 nm) using an external calibration curve (y = 0.0158x + 0.012, R^2^ = 0.986) within the working range of 15–100 μg mL^–1^ in phosphate–borate–citrate buffer (PBC, 10 mM, pH 7.4).

1 $$\mathrm{\%EE}=\left(\frac{{[\mathrm{Eug}]}_{\left(\mathrm{found}\right)}}{{[\mathrm{oil}]}_{\left(\mathrm{inicial}\right)}}\right)\times 100$$

#### 2.3.2 Morphological Characterization of Zein Nanoparticles

The morphology of the NPZ was analyzed using transmission electron microscopy (TEM) on a Morgani 268D instrument (FEI Company) operated at an accelerating voltage of 100 kV. For this analysis, NPZ, NPZ-CO, and NPZ-Eug samples were diluted in deionized water at 10-fold and 5-fold, respectively. Then, 2 μL of each colloidal suspension was carefully deposited onto copper grids (400 mesh) coated with a thin carbon–Formvar film. The grids were placed in a Petri dish containing filter paper and kept in a desiccator to allow solvent evaporation for 24 h at room temperature.^15^

#### 2.3.3 Studies of Colloidal Stability

The colloidal stability of the NPZ was evaluated through three distinct assays, according to the classification of the Brazilian Health Regulatory Agency (ANVISA–Brazil):^16^ *exploratory stabilit*y, *accelerated stability*, and *normal stability*. In the exploratory stability assay, the samples (NPZ, NPZ-Eug, NPZ-CO) were subjected to centrifugation for 90 min at speeds of 5000, 10000, and 15000 rpm. After centrifugation, the colloidal suspension was visually analyzed for appearance, presence of the Tyndall effect, and sediment formation.

For the normal stability assay, the same samples were prepared and stored for 42 days under two distinct conditions: room temperature (∼25–28 °C) and refrigeration (∼5 °C). At weekly intervals, physicochemical parameters were evaluated, including pH (measured with a PHS3B2 pH meter, BEL), turbidity (AP2000 turbidimeter, PoliControl), and encapsulation efficiency (%EE), calculated according to eq 1).

In the accelerated stability assay, refrigerated samples were used as controls, while others were subjected to alternating temperature cycles: 24 h under refrigeration followed by 24 h at room temperature. The same physicochemical parameters mentioned above were monitored throughout the cycles.

#### 2.3.4 *In Vitro* Studies of Eugenol Release Kinetics

The release of eugenol from NPZ-Eug and NPZ-CO was evaluated *in vitro* at 25 °C using phosphate–borate–citrate buffer (PBC, 10 mM, pH 7.4). Aliquots of 5 mL of each sample were placed in SnakeSkin dialysis membranes (3.5 kDa MWCO, 34 mm diameter, ∼15 cm length), with both ends sealed. The membranes were then immersed in 45 mL of PBC buffer and kept under continuous stirring (400 rpm) for 220 min. Every 20 min, 3 mL aliquots of the external medium were withdrawn and replaced with an equal volume of fresh buffer in order to maintain sink conditions in the system. The experiment was conducted in genuine triplicate. The concentration of released Eug was determined by UV–vis molecular absorption spectrophotometry (λ = 280 nm) using an external calibration curve prepared under the same conditions (y = 0.0158x + 0.012, R^2^ = 0.986) with a working range of 15–100 μg mL^–1^. The analytical method used was previously validated and published by our group, confirming its accuracy for determining eugenol concentration.^8^

The release kinetics of the active compound (Eug) were evaluated using six mathematical models: zero-order (eq 2), first-order (eq 3), Hixson–Crowell (eq 4), Higuchi (eq 5), Korsmeyer–Peppas (eq 6), and Weibull (eq 7). The experimental data were fitted to the respective equations, in linear form (eqs 2–4) or nonlinear form (eqs 5–7), using OriginPro 8 software.

2 $$f_{t}=k_{0}t+b$$

3 $$\ln\left(1-f_{t}\right)=-k_{1}t+b$$

4 $${\left(1-f_{t}\right)}^{1/3}=-k_{\mathrm{HC}}t+b$$

5 $$f_{t}=k_{H}t^{1/2}$$

6 $$f_{t}=k_{\mathrm{KP}}t^{n}$$

7 $$f_{t}=1-\exp\left[-t^{\beta }/\alpha \right]$$

where *t* = time, *k* = release rate constant, *f*_*t*_ = cumulative fraction of eugenol released, *b* = linear coefficient of the regression equation, *n* = release exponent characterizing the release mechanism, α = release time scale, and β = curve shape parameter: with β = 1 (case 1) representing an exponential curve; β > 1 (case 2) a sigmoidal curve; and β < 1 (case 3) a parabolic curve, with a steeper initial slope followed by a trend consistent with an exponential function.^17^

The maximum amount of eugenol (Eug) releasable from the nanoparticles was determined by first quantifying the Eug content in the NPZ-Eug and NPZ-CO formulations used in the dialysis assay.^8^ For this purpose, 0.5 mL of the nanoparticle suspension was mixed with 3.5 mL of phosphate–borate–citrate buffer (PBC, 10 mM, pH 7.4). The mixture was incubated for approximately 3 h, with periodic homogenization on a Vortex mixer every 30 min to promote complete diffusion of the active compound into the medium. After the incubation period, samples were subjected to ultrafiltration using Amicon filters (3.4 kDa), and the total concentration of Eug released was determined by UV–vis molecular absorption spectroscopy, as previously described.

### 2.4 Insecticidal Assays against *Sitophilus zeamais*

#### 2.4.1 Rearing of *Sitophilus zeamais*

Adult specimens of *Sitophilus zeamais* were obtained from a laboratory-rearing colony maintained on untreated maize grains under controlled temperature (23–27 °C) and relative humidity (68–82%). The colony was kept in 1 L glass containers sealed with perforated plastic film to allow gas exchange. The maize used as a food substrate was free from insecticide residues and contamination by other insect species.^18^

#### 2.4.2 Evaluation of Direct and Indirect Contact Toxicity

Indirect contact toxicity assays were performed using NPZ, NPZ-Eug, NPZ-CO, Eug, and CO as test treatments. The commercial insecticide K-Othrine (diluted 125-fold to obtain 0.02% v/v deltamethrin) was used as the positive control, while PVA/EtOH solution (0.6% v/v) and distilled water served as negative controls. For each treatment, 600 μL of the respective sample was applied onto 8 cm diameter filter paper discs. The treated discs were then placed inside polystyrene Petri dishes (8.5 cm diameter × 1.5 cm height), whose lids contained a circular opening of 6.5 cm diameter covered with white voile fabric, allowing proper ventilation. After solvent evaporation (∼6 min), ten unsexed *S. zeamais* adults of standardized body size were introduced into each dish. The concentrations of treatments containing oil (CO or Eug) were standardized at 1.5 mg mL^–1^, resulting in a surface dose of 0.018 mg cm^–2^).

Direct contact toxicity assays were performed with the same samples used in the indirect contact tests. Aliquots of 600 μL of each sample were sprayed directly onto ten unsexed adults of *S. zeamais*, previously placed on 8 cm diameter filter paper discs positioned inside the same Petri dishes described above. The concentrations of treatments containing oil (CO or Eug) were maintained at 1.5 mg mL^–1^, corresponding to a dose of about 0.25 mg insect^–1^). Mortality was assessed after 24, 48, and 72 h of exposure. All treatments and controls were carried out with five independent replicates.^19^

The nanoparticle doses used in the biological assays were determined based on two criteria: (i) the maximum oil concentration that maintained colloidal stability and (ii) the largest spray volume that could be applied without impairing respiration or causing mechanical mortality in the insects.

#### 2.4.3 Evaluation of Repellent Effect

Repellency tests were conducted in experimental arenas consisting of three plastic containers (350 mL) symmetrically connected by two cylindrical tubes (10 cm length × 7 mm diameter). The central container was used for insect release, while the two lateral containers, placed on opposite sides, served as choice zones: control (C) and treatment (T) (Figure S1). Twenty unsexed adults of *S. zeamais* were introduced into the central compartment. In one of the lateral containers, 50 g of maize grains homogenized with 500 μL of the samples (NPZ-CO, NPZ-Eug, NPZ, CO, Eug, or PVA/EtOH) were placed, while the other contained 50 g of grains homogenized with 500 μL of distilled water (control). Free oil samples (CO or Eug) were prepared at a concentration of 1.5 mg mL^–1^, corresponding to a dose of 15 mg kg^–1^. Each treatment was carried out with seven replicates, and the repellent activity was determined by counting the number of insects present in each compartment after 3, 6, 12, 24, 48, and 72 h of exposure.^20,21^

#### 2.4.4 Population Development of *S. zeamais* on Treated Maize Grains

The treatments (NPZ, NPZ-CO, NPZ-Eug, CO, and Eug), along with the negative controls (PVA/EtOH and distilled water) and the positive control (deltamethrin), were used to evaluate population development and adult mortality of *S. zeamais*. For this purpose, 250 mL plastic containers (9 cm diameter × 6 cm height) were filled with 100 g of maize grains and 1 mL of the respective sample. Ten unsexed adults of *S. zeamais* were introduced into each container, with five replicates for each experimental condition.

Free oil samples (CO or Eug) were prepared at a concentration of 1.5 mg mL^–1^, resulting in a dose of 15 mg kg^–1^. Population growth and mortality were assessed every 7 days over a total period of 49 days by counting the number of live and dead individuals. At the end of this period, the number of emerged adults was recorded and calculated as the difference between the total number of weevils present and the initial number of individuals used for artificial infestation. Final mortality was expressed as a percentage, based on the ratio of dead individuals to the total population, multiplied by 100.

After 63 days of infestation, the following additional analyses were performed: (a) percentage of damaged grains, determined by the weight difference between intact and damaged grains; (b) grain weight loss, obtained by comparing the mass of the grains before and after exposure to the insects; and (c) grain moisture content, evaluated by weighing 20 g of grains before and after drying in an oven at 105 °C for 24 h. For this last analysis, two replicates were carried out per treatment, including the five treatments (NPZ, NPZ-CO, NPZ-Eug, CO, and Eug), the negative controls (PVA/EtOH and water), and the positive control (deltamethrin).^22^

#### 2.4.5 Statistical Analysis

The data obtained from the bioassays were first evaluated for normality using the Shapiro-Wilk test. Since the results of the direct and indirect contact toxicity assays, mortality, and population development of *S. zeamais* did not follow a normal distribution, they were analyzed using nonparametric methods (Kruskal–Wallis and Dunn tests) at a 1% significance level in R statistical software. These tests were chosen because they are more appropriate for data sets with non-normal distribution and a relatively small sample size (*n* = 5), ensuring reliable comparisons under these experimental conditions. Results were expressed as median values.

Data related to maize grain damage and moisture content showed normal distribution and homogeneity of variances. Therefore, they were analyzed by analysis of variance (ANOVA) using SISVAR statistical software. When significant differences were detected at the 5% level by the F-test, means were compared using the Scott–Knott test, also at the 5% significance level.

For the repellency tests, the data were transformed by √(x+1) before applying ANOVA. When significant differences were observed by the F-test (*p* < 0.05), treatment means were compared using Tukey’s test at the same significance level.

Each bioassay was conducted with five to seven replicates, and each replicate consisted of a group of ten adult *S. zeamais* individuals. This design was adopted to reduce individual variability, as each replicate represents a biological subsample rather than a single insect. In this way, the statistical analysis reflects the mean response of a small population per treatment, providing a more representative estimate of the population mean even with a limited number of replicates. An overview of the statistical methods applied to each parameter is shown in Table S1.

## 3 Results and Discussion

### 3.1 Extraction and Analysis of Essential Oil

The essential oil (CO) from dried flower buds of *Syzygium aromaticum* was extracted by hydrodistillation using a Clevenger apparatus for 300 min. The process yielded 10.3% (w/w), which is consistent with literature reports describing yields between 1% and 14% (w/w) for extractions performed for ∼100 to 240 min using the same technique.^23,24^ The wide variation in yields reported in the literature is mainly attributed to differences in the size of the flower buds used.

Gas chromatography with flame ionization detection (GC-FID) revealed four main peaks (Figure S2), the two most intense corresponding to eugenol (4-allyl-2-methoxyphenol, peak a) and eugenyl acetate (peak c). Eugenol is a phenolic compound of the phenylpropanoid class. β-Caryophyllene (peak b), a bicyclic sesquiterpene, commonly occurs with its oxidation product, β-caryophyllene oxide (peak d).^25^ Eugenyl acetate, in turn, is an aromatic ester derived from eugenol.^26^ Collectively, these organic compounds are the main contributors to the biological properties of CO, such as repellent and insecticidal effects.^27,28^

The compounds identified by GC-MS in the CO sample were confirmed by comparing their mass spectra with those in the instrument’s database (NIST 11) (Table S2) and with literature data.^14^ The eugenol content in CO was quantified by UV–vis electronic spectroscopy (λ = 280 nm). Using the calibration curve of the eugenol standard (y = 0.017x + 0.0265), the eugenol (Eug) concentration in the sample was determined as 5700 μg mL^–1^. This concentration corresponds to 78.60% (v/v) of CO, a result close to that obtained by GC-FID analysis based on the eugenol peak area in the chromatogram (79.32% v/v, Table S2).

Alimia et al. (2023),^29^ using the same hydrodistillation technique to extract CO, identified seven components that together accounted for 100% (v/v) of the oil: eugenol (97.66%), eugenyl acetate (0.97%), caryophyllene (0.69%), isoeugenol (0.32%), caryophyllene oxide (0.13%), thymol (0.11%), and α-humulene (0.09%).

### 3.2 Preparation and Physicochemical Characterization of Nanoparticles

Zein nanoparticles were obtained using the nanoprecipitation method previously described by our group.^8^ In this method, NPZ are formed through a rapid diffusion of ethanol (organic phase) into the aqueous phase. This diffusion increases the polarity of the medium surrounding zein, leading to the spontaneous aggregation of its molecules while simultaneously incorporating the active compound present in the reaction medium. The resulting particles have sizes within the nanometric range and are therefore classified as colloidal dispersions.^30^

The formation of zein nanoparticles (NPZ) was quickly confirmed by a visible change in the solution, which turned from translucent to opalescent, a characteristic appearance of colloidal systems (Figure 1a). The colloidal nature of the suspension was further confirmed by the observation of the Tyndall effect, evidenced by the scattering of a laser beam as it passed through the suspension (Figure 1b).

**Figure 1:**
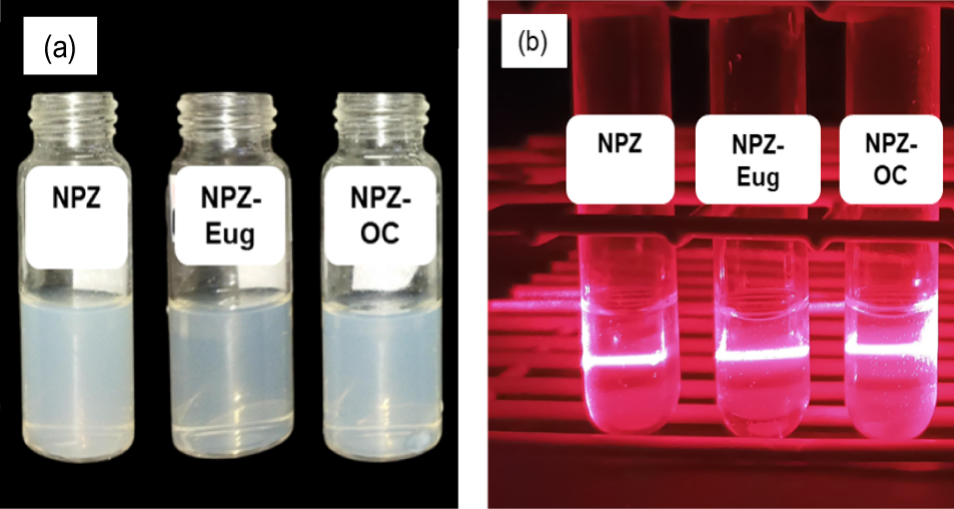
Nanoparticulate systems: (a) vials containing freshly prepared nanoparticle suspensions; (b) Tyndall effect observed in nanoparticle dispersions.

The particle size distribution, polydispersity index (PDI), and zeta potential of the three nanoparticle formulations are presented in the Supporting Information (Figure S3), providing additional evidence of their colloidal stability. The size distribution exhibited a single Gaussian peak with hydrodynamic diameters (Z-average) and PDI values consistent with typical nanoparticulate systems: NPZ (202 nm/0.195), NPZ-Eug (283 nm/0.310), and NPZ-CO (313 nm/0.236). PDI values below ∼0.3 indicate a narrow and homogeneous size distribution of the particles.^77^ The hydrodynamic diameters of the loaded nanoparticles (NPZ-Eug and NPZ-CO) were larger than those of unloaded NPZ, as expected, since the incorporation of active compounds into polymeric matrices typically increases particle size due to the inclusion of additional mass and possible structural rearrangements.^78^ The zeta potential values of NPZ (−31.2 mV), NPZ-Eug (−19.5 mV), and NPZ-CO (−15.7 mV) fall within the range associated with good electrostatic stability. The negative surface charge is attributed to the anionic amino acid residues of zein, as PVA acts as a nonionic stabilizer. The decrease in zeta potential upon incorporation of eugenol or clove oil suggests partial surface coverage of zein by these hydrophobic molecules, which reduces the net surface charge.

Morphological characterization by transmission electron microscopy (TEM) revealed predominantly spherical and irregular nanoparticles, with diameters below 100 nm and the presence of aggregation points (Figure 2a). The image obtained for NPZ-Eug also showed particles within the nanometric scale, with morphology consistent with the controlled release of the active compound (Figure 2b). The average diameter of the nanoparticles obtained in TEM is consistent with values reported in the literature,^31,32^ reinforcing the reliability of the results. However, the particle sizes observed in the microscopy images were considerably smaller compared to the average diameter obtained by Dynamic Light Scattering (DLS) (Figure S3). This difference can be explained by the specific characteristics of the techniques: while DLS measures the hydrodynamic diameter of nanoparticles in suspension, microscopy analysis involves a prior dehydration process, which leads to particle shrinkage and, consequently, an apparent reduction in size.^33^

**Figure 2:**
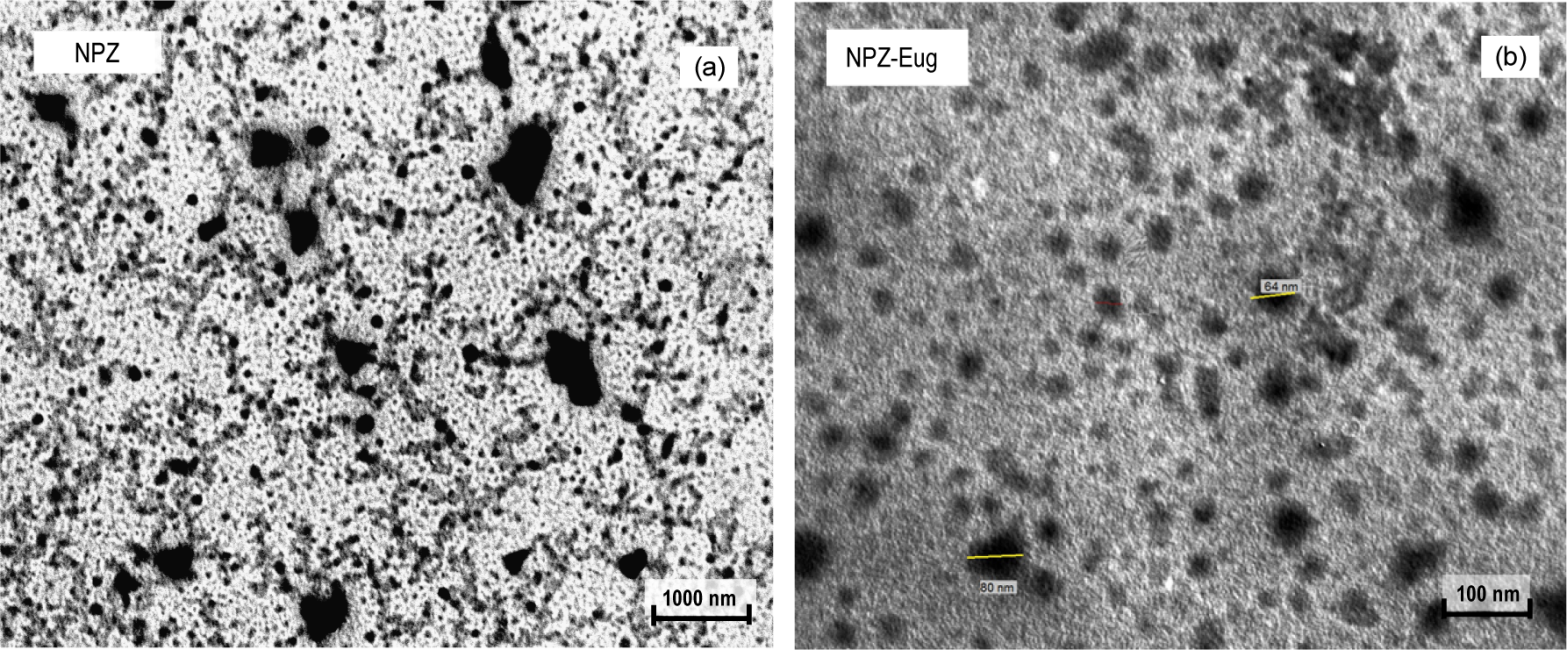
Representative transmission electron microscopy (TEM) image of nanoparticles showing particles with diameters below 100 nm: (a) zein nanoparticles (NPZ); (b) zein nanoparticles loaded with eugenol (NPZ-Eug).

### 3.3 Studies of Colloidal Stability

For commercial applications, it is essential that the formulations produced maintain their stability throughout the product’s shelf life. Therefore, it is crucial to evaluate their long-term stability under simulated environmental conditions.^34^ Accordingly, the nanoparticulate systems produced in this study (NPZ, NPZ-CO, NPZ-Eug) were subjected to different conditions and simulated environmental factors through exploratory, accelerated, and normal stability tests. Over 42 days of monitoring, no signs of nanoparticle coalescence or sedimentation were observed, indicating good stability of the systems evaluated.

#### 3.3.1 Exploratory Stability

After centrifugation, nanoparticle dispersions may exhibit instability phenomena such as sedimentation, flocculation, or emulsification.^35^ This occurs because centrifugation simulates an increased gravitational force, which in turn enhances nanoparticle mobility and accelerates potential instabilities.^16^ In this context, this exploratory test was qualitatively designed to identify the onset of physical instabilities rather than quantify sedimentation, and to determine the minimum centrifugation speed capable of inducing sedimentation of the larger particles.

Sediment formation with an orange coloration was observed only at 10,000 rpm and 15,000 rpm after 90 min of centrifugation. This sedimented material may be related to the presence of larger particles, which are more susceptible to centrifugal force. However, even with sedimentation at these speeds (10,000 and 15,000 rpm), the suspensions retained their original organoleptic characteristics, showing only a slightly less opalescent appearance compared to the noncentrifuged samples. In addition, no coalescence was observed at any of the three tested speeds, and the Tyndall effect confirmed the presence of nanoparticulate material in suspension under all evaluated conditions. These results indicate that centrifugation speeds above 10,000 rpm can promote sedimentation of larger particles without compromising the integrity of the colloidal suspension.

#### 3.3.2 Normal Stability

Throughout the evaluation period, the nanoparticulate systems remained stable, with no changes in odor, precipitate formation, or visible phase separation. Similar results have been reported in previous studies with NPZ formulations containing carvacrol, which remained stable for 21 days at temperatures ranging from 4 to 25 °C.^35^

The pH and turbidity of the samples were monitored over a 42-day period as part of the stability assessment. Monitoring pH is important because fluctuations may indicate the release of encapsulated active compounds,^36^ while turbidity reflects the degree of particle aggregation in suspension, i.e., higher turbidity corresponds to greater aggregation.^37^ In the systems analyzed, pH increased slightly from 3.4 to 4.3 during the first 14 days of storage (Figure 3). From days 14 to 28, NPZ-CO and NPZ-Eug maintained stable pH values under both storage conditions. After this period, a gradual decrease in pH (from 4.4 to 3.3) was observed, whereas in NPZ, the decline began as early as day 14. Despite these small variations, the observed pH changes are considered within the normal range.

**Figure 3:**
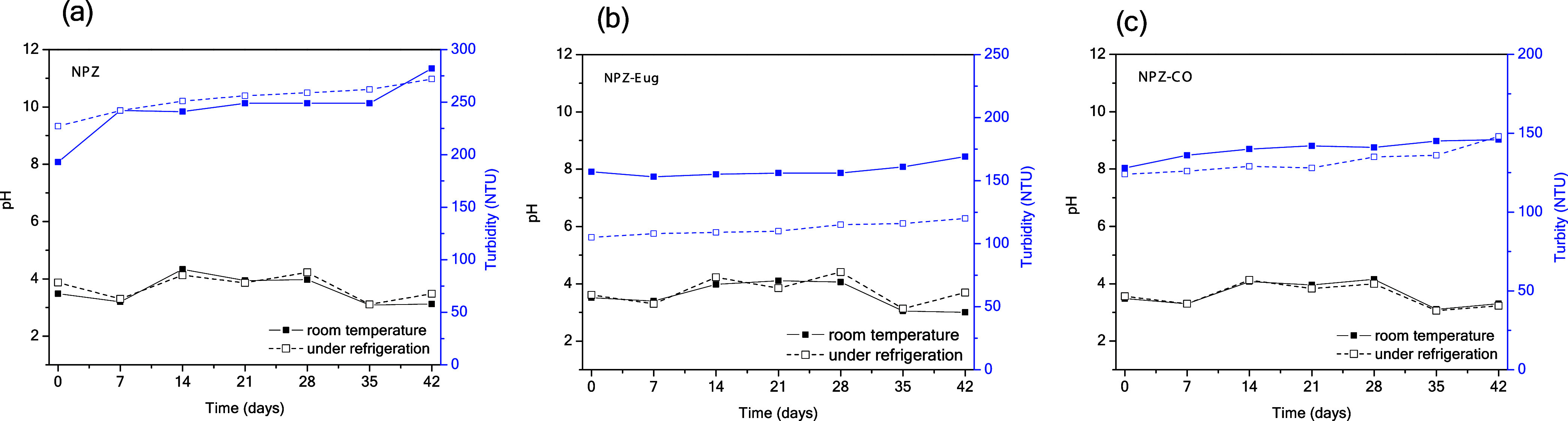
Evaluation of pH and turbidity of nanoparticles during 42 days of normal stability tests: (a) zein nanoparticles (NPZ); (b) zein nanoparticles loaded with eugenol (NPZ-Eug); (c) zein nanoparticles loaded with clove essential oil (NPZ-CO).

For NPZ-CO and NPZ-Eug samples, whether stored under refrigeration or at room temperature, turbidity showed only a slight increase during the monitoring period (Figure 3b and c), with a variation of about 20 NTU. Such a change is considered acceptable and does not indicate nanoparticle instability. In contrast, NPZ (Figure 3a) exhibited a much sharper increase in turbidity, close to 150 NTU, suggesting a higher tendency toward instability. This behavior may be associated with the absence of the active compound, which appears to help maintain particle integrity and dispersion, making NPZ less stable than NPZ-Eug and NPZ-CO. Similar behavior has been reported in the literature for others NPZ.^8,38^ The greater stability of the encapsulated systems can be attributed to stronger interactions between the active compound, the polymeric matrix, and the surfactant, which favor the formation of smaller particles with a more homogeneous distribution and a more stable charge balance.^8^

The analysis of encapsulation efficiency (%EE) over time (Figure S4) revealed significant differences among the systems evaluated and the storage conditions. At the initial time point, NPZ-Eug showed higher performance at room temperature (87%) compared to refrigeration (83%). After 42 days, these values slightly decreased to 83% for samples stored at room temperature and 78% under refrigeration. Even with this reduction, the values remained above 70%, which is considered acceptable for nanoparticulate systems,^36^ indicating good stability throughout the period. In contrast, NPZ-CO presented lower initial %EE values: 69% at room temperature and 73% under refrigeration. By the end of the 42 days, these rates dropped to 62% and 68%, respectively, showing a more pronounced loss of efficiency in these systems. Overall, NPZ-Eug maintained higher %EE than NPZ-CO throughout the monitoring period. Furthermore, refrigeration proved more effective in preserving %EE, especially in NPZ-CO, highlighting the importance of storage conditions in prolonging the stability of these systems.

The %EE of the systems studied (NPZ-Eug and NPZ-CO) showed a gradual reduction over time, as expected, since the active compound incorporated into the polymeric matrix is volatile and tends to be progressively released, although at a slower rate (Figure S4). Similar results were reported by Pascoli et al. (2020)^39^ for NPZ encapsulating neem oil, as well as by Oliveira et al. (2018)^11^ when studying NPZ systems containing geraniol and R-citronellal. High %EE values of botanical compounds and their components are essential for field applications, as they ensure that most of the compounds remain protected within the matrix, preventing premature degradation.^12^

#### 3.3.3 Accelerated Stability

The accelerated stability study was conducted with two sets of each nanoparticulate system (NPZ, NPZ-CO, NPZ-Eug). Samples stored under refrigeration were defined as “control,” while those exposed to alternating temperature cycles were referred to as “cycle” (Figure S5). During the 42-day period, the colloidal suspensions remained visually stable, similar to the results presented in the normal stability study. Nevertheless, slight oscillations in physicochemical parameters were observed, especially in the NPZ. Overall, the pH of the encapsulated systems (NPZ-CO and NPZ-Eug) ranged from 3.5 to 4.5, a range consistent with zein stability, while NPZ showed variations from 3.5 to 5.0. Turbidity fluctuated around 40 NTU for all systems throughout the evaluation period.

During monitoring, the pH of the NPZ subjected to alternating temperature cycles gradually increased, reaching 5.0 after 21 days. This value is close to the isoelectric point of zein (pH 6.2), which may indicate the onset of colloidal instability. This change is attributed to possible degradation of the polymeric matrix, involving chain relaxation, bond cleavage, and structural alterations.^36,40^ In contrast, the encapsulated systems maintained pH within a more stable range, suggesting greater resistance to variations imposed by thermal cycles.

With respect to turbidity, all systems showed a slight increase over time, indicating possible gradual aggregation of the nanoparticles. Nevertheless, visual stability was maintained until the end of the monitoring period. The NPZ, particularly under the cycle condition, exhibited an earlier and more pronounced increase in turbidity, reinforcing signs of instability from the first days. In contrast, the NPZ-CO and NPZ-Eug samples displayed more stable behavior, corroborating the results obtained in the normal stability assay.

### 3.4 *In Vitro* Studies of Eugenol Release Kinetics

The controlled release profile of eugenol from NPZ-CO and NPZ-Eug was investigated in buffered medium (pH 7.4) in order to simulate conditions compatible with agricultural applications and under sink conditions to prevent saturation of the eugenol released into the external medium. The total Eug content and its release over time were determined by UV–vis spectrophotometry using an external calibration curve, and the data were expressed as cumulative eugenol release percentage (Figure 4).

**Figure 4:**
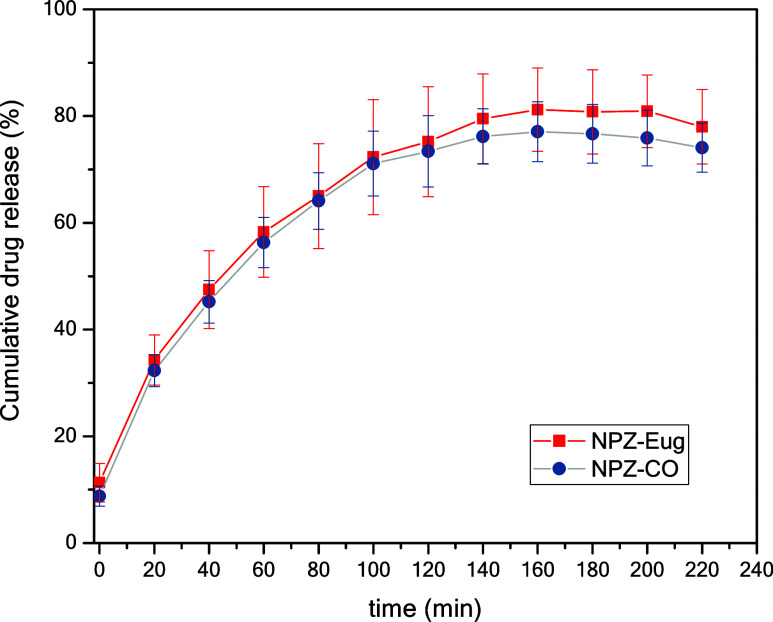
*In vitro* release profiles of NPZ-Eug and NPZ-CO in phosphate–borate–citrate (PBC) buffer (pH 7.4).

In both evaluated systems (NPZ-CO and NPZ-Eug), a biphasic release profile was observed, characterized by an initial rapid phase followed by a more controlled phase, a pattern typically reported for nanoparticulate delivery systems.^41^ The initial stage corresponded to the burst release phenomenon, in which approximately 30% of the eugenol was rapidly released within the first 30 min. This effect is attributed to the fraction of the active compound located at or near the nanoparticle surface, enabling its rapid diffusion into the medium. Subsequently, the system transitioned into a prolonged release phase extending to around 120 min, during which the cumulative release gradually increased to about 75%. Beyond this period, the curve moved into a plateau, reaching approximately 80% around 220 min, indicating that the system had entered its saturation region. This biphasic release pattern is highly desirable for agricultural applications, since a substantial portion of the active compound is delivered shortly after application, followed by sustained release, thereby reducing the need for frequent reapplications and minimizing losses due to volatilization or compound degradation.

Smart polymeric nanoparticles, also referred to as stimulus-responsive or environmentally sensitive delivery systems, can modulate the release of bioactive compounds in response to external triggers such as pH, light, or temperature.^45^ In pH-responsive matrices, the presence of ionizable functional groups (e.g., −COOH and −NH_2_) enables swelling or contraction of the polymer network, generating conformational changes that directly influence release kinetics.

In zein-based nanoparticles, pH modulation becomes particularly relevant because this protein is amphiphilic and enriched with nonpolar amino acids (tryptophan, proline, phenylalanine, leucine), whose electronic properties contribute to the matrix’s intermolecular interactions and structural organization.^46^ The predominant fraction, α-zein, has an isoelectric point around pH 6.2;^47^ thus, under the neutral conditions used in the release assays (pH 7.4), its amino and carboxyl groups remain largely deprotonated, imparting a net negative surface charge to the particles.^48^ This physicochemical environment weakens electrostatic attraction between the matrix and the active compound, favoring faster diffusion from the particle surface and contributing to the rapid kinetic profiles observed for both systems, which approached their plateau within approximately 220 min (Figure 4).

This pattern aligns with previous reports of eugenol and other hydrophobic bioactives encapsulated in protein-based carriers (Table S3). Similar biphasic profiles have been observed using rice-protein nanoparticles, where a rapid initial release was followed by a slower sustained phase,^42^ as well as in zein nanoparticle systems in which an early fast diffusion step was followed by a gradual plateau at longer incubation times.^54^ Overall, these findings indicate that an initial fast release followed by diffusion-controlled release is a characteristic behavior of protein-based delivery systems containing essential oils.

Comparable trends have been reported for other phytochemicals encapsulated in zein-based systems. For example, naringin-loaded modified zein nanoparticles showed a distinct biphasic pattern, with rapid initial release followed by a slower diffusion-controlled phase.^43^ A similar behavior was observed for lutein, where an early fast release, associated with matrix swelling and surface-associated compounds, preceded a more gradual diffusion stage.^49^ These findings support the interpretation that protein-based nanocarriers commonly exhibit fast initial diffusion of surface-associated molecules followed by a slower release governed by matrix–compound interactions.

Different mathematical models were applied to describe the kinetic behavior of eugenol release from NPZ-CO and NPZ-Eug, including zero-order (eq 2), first-order (eq 3), Hixson–Crowell (eq 4), Higuchi (eq 5), Korsmeyer–Peppas (eq 6), and Weibull (eq 7) models. The analysis of these models allows the interpretation of the possible mechanisms involved in the process, such as adsorption and diffusion of the active compound through the polymeric matrix, particle erosion, combined processes (diffusion and erosion), as well as potential contributions from polymer degradation by chemical mechanisms. The proper selection and application of the mathematical model make it possible to evaluate the release rate, identify inflection points in the dissolution profile, and understand the predominant mechanisms over time.^50^

The kinetic parameters obtained from linear and nonlinear regressions, including rate constants, slope and intercept coefficients, and correlation coefficients (R^2^), are presented in Table 1. The R^2^ value was used as an indicator of the goodness of fit, with values closer to 1.0 indicating a stronger correlation between the experimental data and the proposed model.^51^

**Table 1:** Kinetic Parameters from Model Fitting for NPZ-Eug and NPZ-CO Samples

Model Kinetics	Fitted Parameters^a^	NPZ-Eug	NPZ-CO
Zero Order	R^2^	0.801	0.700
	*k*_0_ (min^–1^)	0.0031 ± 0.0005	0.0026 ± 0.0005
	b	0.31 ± 0.05	0.33 ± 0.06
First Order	R^2^	0.924	0.794
	*k*_1_ (min^–1^)	0.0079 ± 0.0007	0.0059 ± 0.0009
	b	0.33 ± 0.08	0.41 ± 0.11
Hixson–Crowell	R^2^	0.892	0.768
	k_HC_ (min^–1^)	0.0019 ± 0.0002	0.0014 ± 0.0002
	b	0.11 ± 0.02	0.13 ± 0.03
Higuchi	R^2^	0.914	0.907
	k_H_ (min^–0.5^)	0.065 ± 0.003	0.060 ± 0.003
Korsmeyer–Peppas	R^2^	0.935	0.939
	k_KP_ (min^–n^)	0.122 ± 0.036	0.132 ± 0.037
	k_KP_ (h^–n^)	0.553 ± 0.032	0.533 ± 0.230
	n	0.37 ± 0.06	0.33 ± 0.06
Weibull	R^2^	0.943	0.950
	α (min)	15.9 ± 7.0	13.6 ± 5.5
	β	0.63 ± 0.10	0.58 ± 0.09

a Units were assigned according to the dimensional form of each kinetic equation.

Among the six mathematical models evaluated, two stood out with the best fit to the experimental data: Korsmeyer–Peppas (R^2^ > 0.93) and Weibull (R^2^ > 0.94) for both nanoparticulate systems (NPZ-Eug and NPZ-CO) at pH 7.4 (Figure S6).

The Korsmeyer–Peppas model is widely applied in the analysis of controlled release systems, particularly during the initial stages of release, as it enables identification of the predominant mechanism through the diffusion exponent (*n*). For spherical matrices, *n* values below 0.45 are characteristic of Fickian diffusion. The values obtained in this study, *n* = 0.37 ± 0.06 (NPZ-Eug) and *n* = 0.33 ± 0.06 (NPZ-CO), fall within this range, indicating that diffusion is the dominant mechanism govering the initial release phase. The kinetic constants (*k*_KP_ = 0.553 ± 0.032 min^–n^ for NPZ-Eug and 0.533 ± 0.230 min^–n^ for NPZ-CO) were also comparable between formulations, suggesting similar release rates under the tested conditions. In line with this, these values are of the same order of magnitude as those reported by da Rosa et al. (2015) for zein nanoparticles loaded with other essential oils, such as thymol (k = 0.287 h^–n^) and carvacrol (k = 0.140 h^–n^), reinforcing the consistency of the observed release behavior.^5^ Additional kinetic parameters from different eugenol- and essential-oil-based systems are summarized in Table S3.

On the other hand, the Weibull model, an empirical approach commonly applied to describe release and dissolution profiles, yields very similar α and β parameters for both systems (NPZ-Eug: α = 15.9 ± 7.0 min; β = 0.63 ± 0.10; NPZ-CO: α = 13.6 ± 5.5 min; β = 0.58 ± 0.09). In this model, α parameter reflects the characteristic time of the release process, and in our case, the comparable values indicate that both formulations released eugenol at similar overall rates. The β parameter provides insight into the mechanism controlling release: values close to 1 are typically associated with first-order behavior; β < 1 denotes a release profile dominated by Fickian-type diffusion and commonly accompanied by an initial rapid phase (burst release); whereas β > 1 reflects more complex kinetics involving simultaneous processes such as diffusion, swelling, erosion, or polymer chain relaxation.^52^

In summary, the Korsmeyer–Peppas and Weibull models provide a coherent interpretation of the release mechanism. The diffusion exponents (*n* < 0.45) indicate Fickian diffusion as the dominant process, while the Weibull β < 1 values capture the initial burst followed by a slower diffusion-controlled phase. This agreement between the two models reinforces that the matrix undergoes no significant erosion under the assay conditions and that the rapid early release corresponds to the fraction of eugenol located near or at the nanoparticle surface.

### 3.5 Bioactivity Assays

#### 3.5.1 Evaluation of Direct and Indirect Contact Toxicity

The insecticidal effect was assessed using two application methods: direct contact and indirect contact. In the direct contact assays, the treatment samples and the controls (positive and negative) were applied directly onto the insects. In the indirect contact assays, the samples were applied to the surface onto which the insects were subsequently introduced.

Mortality assessments were carried out at 24, 48, and 72 h using five replicates, each containing ten unsexed adult insects. The administered dose in the indirect (0.018 mg cm^–2^) and direct contact assay (approximately 0.25 mg insect^–1^) showed no insecticidal effect on *S. zeamais*, as all insects remained alive throughout the monitoring period. It is important to note that the doses applied in both assays were determined empirically, considering the maximum feasible concentration of the colloidal suspensions and the experimental setup employed. The final values were calculated based on the nanoparticle suspension concentration, the applied volume, the treated surface area, and the number of insects per Petri dish. These parameters were optimized to ensure a uniform sample distribution while avoiding mortality due to excess liquid or substrate saturation. Because of these experimental constraints, testing higher doses was not feasible. Therefore, only a single concentration was evaluated in bioactivity assays.

The treatments (NPZ-Eug, NPZ-CO, NPZ, CO, and Eug) were compared with the negative controls (water and PVA+ethanol) using the Kruskal–Wallis test at a 1% significance level (Table S4). This nonparametric statistical method was chosen to analyze differences among independent groups for a single continuous variable, since the Shapiro–Wilk test indicated that the data did not follow a normal distribution.^55^ Subsequently, the same treatments and negative controls were compared with the standard insecticide (deltamethrin) using Dunn’s test, also at a 1% significance level (Table S4).

The Kruskal–Wallis test indicated that the active compounds, whether in free form (CO and Eug) or incorporated into zein (NPZ-CO and NPZ-Eug), showed no toxic effect at the administered dose (0.018 mg cm^–2^), as no significant differences were observed compared with the negative controls (water and PVA+EtOH) in controlling *S. zeamais* (Table S4). It is important to emphasize that the dose or initial concentration used in the assays could not be increased, since the applied volume could not be expanded without compromising the gas exchange of insects. Larger volumes could obstruct the spiracles with excess liquid, impairing respiration and interfering with the experimental outcome. Moreover, the nanoparticles were formulated using the previously optimized maximum loading of CO or Eug. This strategy ensured that the samples reached the highest possible encapsulation potential for each active compound while maintaining the limits required for system stability and efficiency.^8^

When Dunn’s test was applied to compare the treatments (NPZ-Eug, NPZ-CO, NPZ, CO, and Eug) and negative controls (PVA+EtOH and water) with the insecticide (positive control), a significant difference was observed, indicating that the insecticide killed more insects than the tested treatments (Table S4). This analysis reinforces the efficacy of the conventional insecticide compared to the alternative treatments studied, which did not show relevant toxicity under the tested conditions. Furthermore, the positive control played a key role in validating the assay, ensuring that the method used was sensitive enough to detect toxic effects when present, thereby strengthening the reliability of the results obtained.

Previous studies have demonstrated insecticidal activity of CO and Eug against *S. zeamais*, but only when applied at doses considerably higher than those used here. For instance, CO showed toxicity at 2.1 μL cm^–2^,^56^ and Eug required concentrations around 5.7 μL mL− 1 ^57^ or topical doses above 47.6 μg mg^–1^ per insect to produce clear toxic effects against *S. zeamais*.^58^ These values are substantially greater than the conditions adopted in our formulation and bioassays, which likely explains the absence of toxic effects observed in the present study.

A related study using *Drosophila melanogaster* tested CO, Eug, and their zein-based nanoparticles at 0.55 mg/mL and found enhanced toxicity after nanoencapsulation. Although the applied volume and effective dose per area were not reported, the lethality ranking was NPZ-CO > NPZ-Eug > CO, while free Eug showed no mortality. Nanoencapsulated samples also caused stronger behavioral impairment, indicating an improvement in bioactivity due to encapsulation.^7^

The absence of insecticidal activity observed here for both free and encapsulated samples is most likely due to the relatively low dose applied and the limited effective exposure of insects to the active compounds. Under the experimental conditions, the application volume had to be minimized to avoid mortality by drowning, which likely reduced both the contact surface and the amount of eugenol available for interaction. Together, these factors suggest that the concentration and exposure conditions used in this study were below the threshold required to produce lethal effects in *S. zeamais*. This reinforces the need for future studies to optimize both dosage and formulation parameters to achieve more pronounced biological effects.

#### 3.5.2 Evaluation of Repellent Effect

The repellency test was conducted to evaluate the effect of the treatments (NPZ-Eug, NPZ-CO, NPZ, CO, and Eug) on the behavior of *S. zeamais* at 3, 6, 12, 24, 48, and 72 h. In this assay, insects could freely move between two arenas: one containing maize grains treated with the formulations and the other containing maize grains treated with water (negative control). Repellency was assessed by monitoring whether insects preferentially migrated toward the untreated grains.

Table S5 presents the mean values and standard deviations for each treatment and time point, which are graphically represented in Figure 5. The corresponding ANOVA results (Table S6) indicate that significant differences among the three choice zones occurred for NPZ-Eug, NPZ-CO, NPZ, and Eug at 3, 6, 24, 48, and 72 h, whereas CO and PVA/EtOH showed significant differences only at 24, 48, and 72 h.

**Figure 5:**
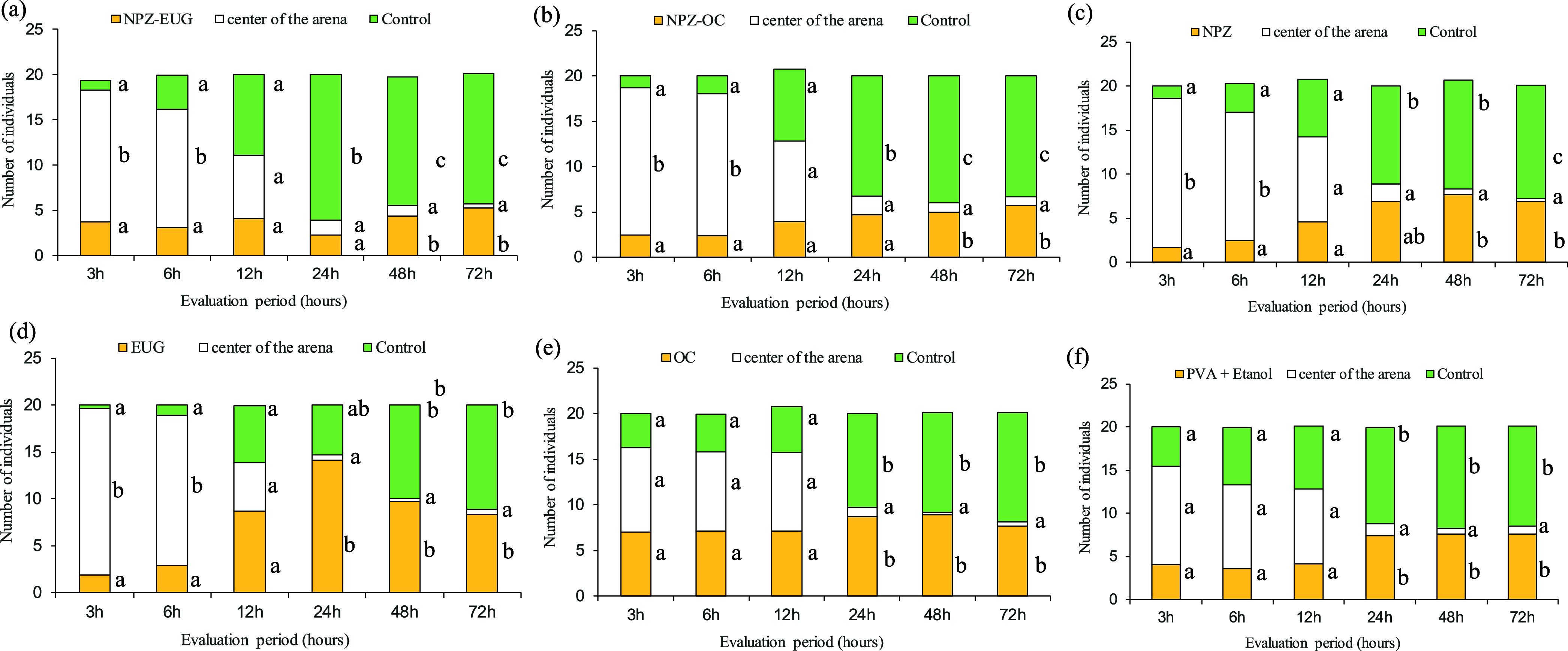
Average preference of *S. zeamais* at 3, 6, 12, 24, 48, and 72 h after application of different samples in choice arenas: (a) zein nanoparticles loaded with eugenol (NPZ-Eug); (b) zein nanoparticles loaded with clove essential oil (NPZ-CO); (c) zein nanoparticles (NPZ); (d) eugenol (Eug); (e) clove essential oil (CO); (f) poly­(vinyl alcohol) + ethanol (PVA+EtOH). Means followed by the same letter within each evaluation period do not differ significantly by Tukey’s test at the 5% significance level. Means were back-transformed for presentation.

The early variations (3–6 h) likely reflect natural insect movement rather than a treatment effect. This interpretation is consistent with the behavioral patterns observed in Figure 5. From 12 h onward, insect displacement increased, but no differences from the control were detected. Clear treatment effects emerged only after 24 h, especially for NPZ-CO and NPZ-Eug, when insects increasingly avoided the treated grains. This time-dependent avoidance reinforces the repellent effect of the nanoparticle formulations under prolonged exposure.

The free CO and Eug samples showed no repellent effect at the tested dose (15 mg kg^–1^). This behavior may be related to the high volatility of these compounds,^27^ since both CO and Eug exhibit low stability when exposed to light, heat, and humidity.^59^ The repellent effect was only observed in the NPZ-CO and NPZ-Eug samples under the same experimental conditions (∼26 °C and relative humidity between 68 and 82%). This result is attributed to the greater stability provided by incorporating CO and Eug into zein nanoparticles, which preserved their repellent properties and enhanced their repellent activity over time.^60^

Thus, the insects exhibited a behavioral response by migrating to the farthest container (control) from the toxic environment containing the NPZ-CO and NPZ-Eug samples as soon as the presence of the active compound was detected (Figure 5). This reaction characterizes a repellent effect, typical of insects exposed to unfavorable conditions.^61^ However, a detailed understanding of the physiological and biochemical mechanisms underlying this behavioral response requires further specific investigations.^62^

Interestingly, the treatment with the NPZ showed a significant difference compared with the control after 72 h (Figure 5). This behavior was unexpected, since zein is a prolamin extracted from maize kernels,^63^ the main food source of the insect studied (*S. zeamais*). Moreover, zein is recognized as nontoxic and is classified by the Food and Drug Administration (FDA) as a safe natural polymer, with approved applications in the pharmaceutical and food industries.^8,64^ To date, and based on the available literature, there is no evidence that zein possesses repellent activity against *S. zeamais*; thus, the effect observed in this study was unexpected and may warrant further investigation to be fully understood.

In the study by Eesiah et al. (2022),^79^ repellency of free essential oils (cinnamon, clove, orange terpenes, thyme, and oregano) against *S. zeamais* was assessed using a four-choice olfactometer composed of a central release chamber and four treated or control arms. Essential oils were tested at concentrations ranging from 1–15% in 10% DMSO, while the remaining arms served as solvent and untreated controls. The authors reported that repellency varied among oils, with cinnamon oil showing the strongest response across concentrations, whereas thyme and clove oils displayed lower efficacy. Notably, combining cinnamon and clove oils resulted in an antagonistic effect, reducing repellency relative to the individual oils.

Using a leaf-disk bioassay, Oliveira et al. (2019)^54^ evaluated the repellency of botanical mixtures containing geraniol combined with either eugenol or cinnamaldehyde against *Tetranychus urticae*. Free oils produced a strong initial repellent effect; however, only the nanoencapsulated formulations maintained the repellency over time, remaining effective for up to 7 days. Overall, zein-based nanoparticles resulted in higher and more persistent repellency than the nonencapsulated mixtures.

Yeguerman et al. (2024)^44^ assessed the repellency of oregano and laurel essential oils, in both free and PEG-encapsulated forms, against *Sitophilus oryzae* and *Lasioderma serricorne* using a two-choice filter-paper arena. Samples were applied at LC_50_ levels (40–300 μg cm^–2^ depending on oil and species), and insect behavior was quantified through metrics such as walking speed, distance traveled, and movement time. Free oils produced only short-term repellency, whereas their nanoencapsulated counterparts prolonged the avoidance response, up to 60 h for oregano oil and 48 h for laurel oil. Overall, encapsulation markedly enhanced both repellency duration and behavioral effects.

Taken together, these findings reinforce that nanoencapsulation plays an essential role in preserving the physicochemical integrity of volatile botanical compounds and sustaining their biological functionality. By reducing evaporation and slowing degradation, the zein nanoparticles enabled a gradual and more persistent release of the active compounds, allowing a clear repellent response at a dose where the free oils showed no detectable effect.

#### 3.5.3 Population Development of *S. zeamais* on Treated Maize Grains

The evaluation of insect population development on treated maize grains was conducted over a period of 49 days. During the first 21 days, population stability was observed, indicating an adaptation phase of the insects to the new environment. After this period, an increase in insect numbers was recorded, showing a population growth pattern that followed a quadratic equation. Figure 6 presents the mathematical equation describing this growth, with R^2^ values >0.93 for all treatments and controls (negative and positive).

**Figure 6:**
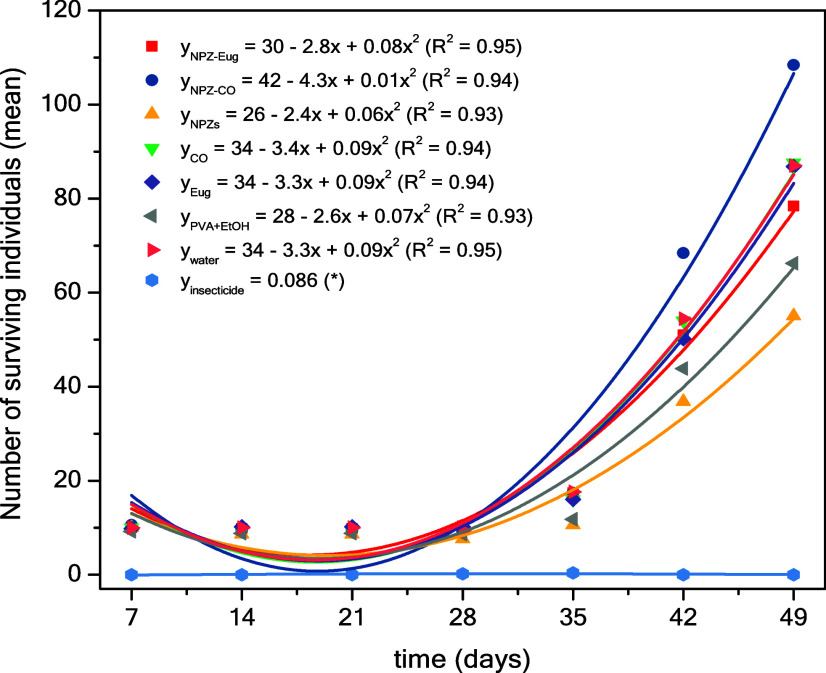
Population growth of *S. zeamais* during the storage period (up to 49 days) of maize grains treated with different samples: zein nanoparticles loaded with eugenol (NPZ-Eug); zein nanoparticles loaded with clove essential oil (NPZ-CO); zein nanoparticles (NPZ); clove essential oil (CO); eugenol (Eug); insecticide (deltamethrin). (*) Data did not fit a linear equation; the value corresponds to the number of live insects (mean across the entire investigated period).

The Kruskal–Wallis test indicated that the active compounds, both in free form and when incorporated into NPZ, did not show a significant effect in reducing the population of *S. zeamais* at the tested dose (15 mg kg^–1^). The analysis was performed by comparing the treated groups with the negative controls (water and PVA+ethanol). The Dunn post hoc test revealed that only the commercial insecticide (positive control) was effective in reducing insect population growth (Table 2).

**Table 2:** Medians of *S. zeamais* Population Growth during the Storage Period (up to 49 Days) of Maize Grains Treated with Different Samples^a^

Samples	7 days	14 days	21 days	28 days	35 days	42 days	49 days
**NPZ-Eug**	10^a^	10^a^	10^a^	11^a^	12^a^	38^a^	65^a^
**NPZ-CO**	10^a^	10^a^	10^a^	10^a^	20^a^	78^a^	122^a^
**NPZ**	10^a^	8^a^	8^a^	8^a^	10^a^	42^a^	65^a^
**Eug**	10^a^	10^a^	10^a^	11^a^	16^a^	53^a^	88^a^
**CO**	10^a^	10^a^	10^a^	11^a^	15^a^	55^a^	99^a^
**PVA+EtOH**	10^a^	9^a^	9^a^	8^a^	14^a^	39^a^	73^a^
**Water**	10^a^	10^a^	10^a^	10^a^	16^a^	59^a^	94^a^
**Treatments and controls**	10^B^	10^B^	10^B^	10^B^	14^B^	49^B^	78^B^
**Insecticide**	0^A^	0^A^	0^A^	0^A^	0^A^	0^A^	0^A^

a Medians followed by the same lowercase or capital letter, separately, within columns do not differ significantly according to the Kruskal–Wallis and Dunn’s tests, respectively, at the 1% significance level. Samples: zein nanoparticles loaded with eugenol (NPZ-Eug); zein nanoparticles loaded with clove essential oil (NPZ-CO); zein nanoparticles (NPZ); eugenol (Eug); clove essential oil (CO); poly­(vinyl alcohol) + ethanol (PVA+EtOH).

When evaluating insect mortality in contact with treated maize over 49 days (Figure S7), a slight increase in the percentage of dead insects was observed around day 30. This effect may be associated with the natural life cycle of *S. zeamais*, since the same pattern was also observed in the negative controls. Moreover, this minor increase in insect mortality was lower than the rate of population growth and therefore cannot be attributed to any toxic effect of the treatment.

The Kruskal–Wallis test indicated that the active compounds, both in free form and when incorporated into zein, did not exhibit significant toxic effects at the tested dose (15 mg kg^–1^). The analysis was performed by comparing the treated groups with the negative controls (water and PVA+ethanol) (Table S7). Previous studies have shown that higher doses of CO (500 μL kg^–1^) produced a significant toxic effect on *S. zeamais* populations when tested with clove and cinnamon oils.^56^

In addition to evaluating population development and cumulative mortality of *S. zeamais* on treated maize, grain damage and moisture content were also assessed over 63 days. At the end of the assay, three categories of grains were identified: healthy (without signs of damage), perforated (with a round hole extending inward), and with galleries (showing visible larval tunnels without an exit hole) (Figure S8).

Maize grains treated with the formulations (NPZ-Eug, NPZ-CO, NPZ, CO, and Eug) and negative controls (PVA+ethanol and water) showed greater damage compared with those treated with the positive control (insecticide), suggesting that the treatments at the tested dose (15 mg kg^–1^) did not inhibit insect feeding. Analysis of variance using the F-test revealed significant differences between damaged and intact grains (Table S8). The Scott–Knott test (Table 3) confirmed that these significant differences occurred between the tested treatments and the insecticide (positive control).

**Table 3:** Average Results of Damaged and Undamaged Maize Grains Caused by *S. zeamais*, and Grain Moisture Content after 63 Days of Storage Following Treatment with Different Samples^a^

Samples	Damaged grains(g)	Undamaged grains(g)	Moisture content(%)
**NPZ-Eug**	57.36^b^	37.74^b^	13.24^a^
**NPZ-CO**	63.06^b^	30.67^b^	13.17^a^
**NPZ**	47.98^b^	49.86^b^	12.58^a^
**CO**	59.59^b^	35.14^b^	13.33^a^
**Eug**	61.6^b^	32.66^b^	12.81^a^
**PVA+EtOH**	47.10^b^	48.82^b^	13.16^a^
**Water**	53.03^b^	45.25^b^	13.04^a^
**Insecticide**	1.54 ^a^	99.91^a^	12.06^a^

a Means of grain mass and moisture content followed by the same letter do not differ significantly according to Scott–Knott’s test at the 5% significance level. Samples: zein nanoparticles loaded with eugenol (NPZ-Eug); zein nanoparticles loaded with clove essential oil (NPZ-CO); zein nanoparticles (NPZ); Eugenol (Eug); Clove essential oil (CO); poly­(vinyl alcohol) + ethanol (PVA+EtOH).

Analysis of Table 3 indicated that the moisture content of maize grains subjected to the different treatments did not differ significantly from one another. However, maize treated with insecticide showed lower moisture content (12.1%) compared with the other treatments, which can be explained by the reduction in the population of live insects in the maize. This decrease results in less water release from the insects’ respiratory activity.^22^

Despite the increase in *S. zeamais* population, all tested samples as well as the controls, maintained moisture content within the ideal range for maize grains, varying between 12% and 13%^65^ (Table 3). This stability can be attributed to the controlled storage conditions and the experimental environment. The samples were stored in a closed room equipped with a dehumidifier that regulated the relative humidity of the air. In addition, each container had perforations to ensure air circulation, and the small quantities of grain facilitated gas exchange by both the insects and the grains, contributing to the maintenance of appropriate moisture levels.

To better interpret the bioassay results, it is essential to recognize that repellency and population development reflect distinct biological responses under different experimental conditions. The apparent divergence between these outcomes arises from the design of each assay. In the repellency test, insects were free to move between treated (T) and untreated (C) areas (see schematic representation in Figure S1), allowing them to select the environment most favorable for their establishment and activity. In contrast, during the population development assay, insects were confined with the treated grains and had no opportunity to relocate. Under these conditions, they could adapt to the environment or wait until the volatile compounds dissipated before resuming feeding and reproduction. Therefore, the increase in damaged grains and insect population observed in this assay does not contradict the repellency results but rather reflects the difference between a short-term behavioral response and long-term exposure.

## 4 Conclusion

The extraction of clove essential oil from the dried flower buds of *S. aromaticum* yielded 10.3% (w/w), a value consistent with previously reported data. Qualitative analysis identified eugenol and eugenyl acetate as the major constituents of the oil, with variations in composition attributed to seasonal factors. Quantification of eugenol by UV–Vis spectrometry corroborated the GC-FID results, confirming the accuracy of the analysis.

The nanoparticles were successfully prepared by the nanoprecipitation method, yielding stable colloidal suspensions, as visually indicated by color changes and the Tyndall effect and further confirmed by Dynamic Light Scattering (DLS). The size distributions were monomodal and exhibited a Gaussian-shaped distribution, with mean hydrodynamic diameters ranging from 230 to 360 nm and PDI values below 0.3, consistent with homogeneous nanoparticulate systems. The zeta potential ranged from −16 to −30 mV, indicating adequate electrostatic repulsion to support colloidal stability. Transmission Electron Microscopy (TEM) revealed irregular spherical nanoparticles. Centrifugation assays showed that the colloidal suspension remained stable at all tested speeds; however, higher speeds (15,000 rpm) promoted the sedimentation of larger particles, leading to reduced turbidity but preserving colloidal integrity. Suspensions stored under refrigeration demonstrated greater physical stability over 42 days, with NPZ-Eug and NPZ-CO showing higher resistance to temperature variations and higher encapsulation efficiency values (EE > 70%).

The eugenol release study from zein nanoparticles (NPZ-Eug and NPZ-CO), carried out at pH 7.4 to simulate agricultural conditions, revealed a biphasic profile with an initial rapid release phase (burst release) followed by sustained release. Kinetic analysis indicated that the Korsmeyer–Peppas and Weibull models provided the best fit to the experimental data, pointing to Fickian diffusion as the predominant mechanism, with no significant degradation of the matrix. The fast initial release is attributed to the fraction of eugenol positioned at or close to the nanoparticle surface. This profile favors the prolonged bioactivity of the compounds, reducing the frequency of reapplications and improving delivery efficiency.

In the bioassays with *S. zeamais*, neither the free compounds (CO and Eug) nor the encapsulated formulations (NPZ-CO and NPZ-Eug) exhibited significant toxicity at the tested dose, regardless of whether exposure occurred by direct or indirect contact. As a result, the treatments did not influence the population development of the species. In contrast, both encapsulated formulations showed a consistent and measurable repellent effect from 24 h onward, even when applied at a single dose of 15 mg kg^–1^. This behavioral response appears to be associated with the greater physicochemical stability of the active ingredients when incorporated into the zein matrix, which helps preserve the integrity of volatile compounds and sustain their repellent performance over time. Future studies will focus on increasing the loading capacity of the zein nanoparticles, evaluating synergistic interactions with other botanical compounds, and expanding the assays to field-like conditions to better assess the practical applicability of the system.

## Supplementary Material



## Notes

Additional supporting figures and tables are provided, including: schematic representation of the repellency arena; chromatographic profile of clove oil; particle size and zeta-potential distributions; encapsulation efficiency and accelerated stability data; eugenol release profiles and kinetic model fits; mortality, repellency, and grain-damage assessments; and detailed statistical summaries for all biological assays (PDF)
