# Supplementary material for: Clove Oil–Loaded Zein Nanoparticles: Eugenol Release and Repellent Activity against Sitophilus zeamais
Source: ACS Omega. 2026 Feb 3;11(6):9642–56. doi: 10.1021/acsomega.5c09938 (PMC12917797; doi:10.1021/acsomega.5c09938)
Supplement: Supplementary file 1 [file ao5c09938_si_001.pdf]

Electronic Supporting Information

Clove Oil–Loaded Zein Nanoparticles: Eugenol Release and Repellent Activity against *Sitophilus zeamais*

Laurieth Góes de Jesus[a], Railan dos Santos Silva[b], Rosilene Aparecida de Oliveira[a], Carla Fernanda Favaro[a], Carlos Eduardo Pereira[b], Rodrigo Luis Silva Ribeiro Santos[a*]

[a] Departamento de Ciências Exatas, Universidade Estadual de Santa Cruz (UESC), Ilhéus, Brasil.

[b] Centro de Formações em Ciências Agroflorestais, Universidade Federal do Sul da Bahia (UFSB), Itabuna, Brasil.

Figure S1 - Schematic representation of the repellency test arena. The central compartment was used for insect release, while the two lateral compartments contained treated (T) and untreated maize grains (C), allowing *Sitophilus zeamais* to choose between control and treatment areas

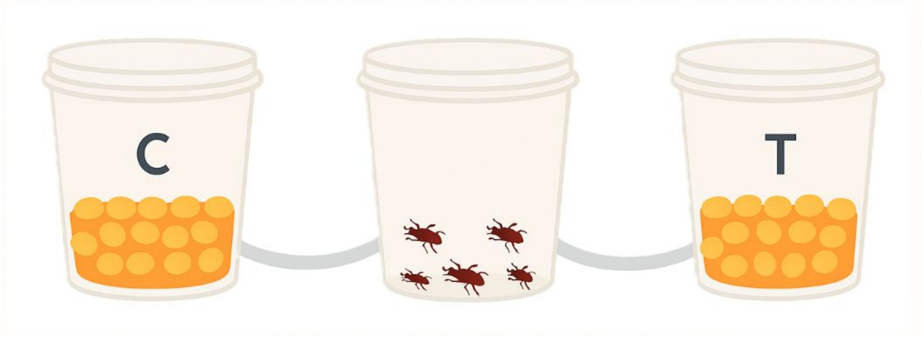

Figure S2 - Chromatogram (GC-FID) of the chemical components present in clove essential oil: eugenol (a), β-caryophyllene (b), eugenyl acetate (c), β-caryophyllene oxide (d)

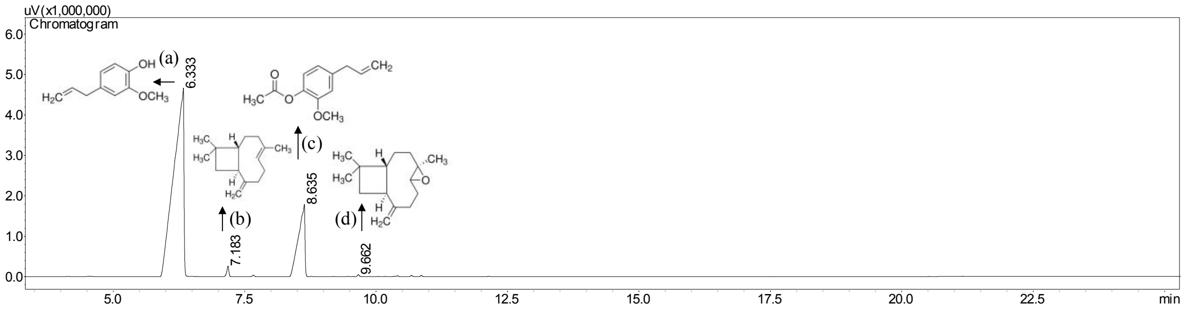

Figure S3 – Particle size distribution (a, c, e) and zeta potential (b, d, f) of zein nanoparticles (NPZ), zein nanoparticles loaded with eugenol (NPZ-Eug), and zein nanoparticles loaded with clove essential oil (NPZ-CO). Data represent typical distributions obtained by dynamic light scattering (DLS) and electrophoretic light scattering (ELS)

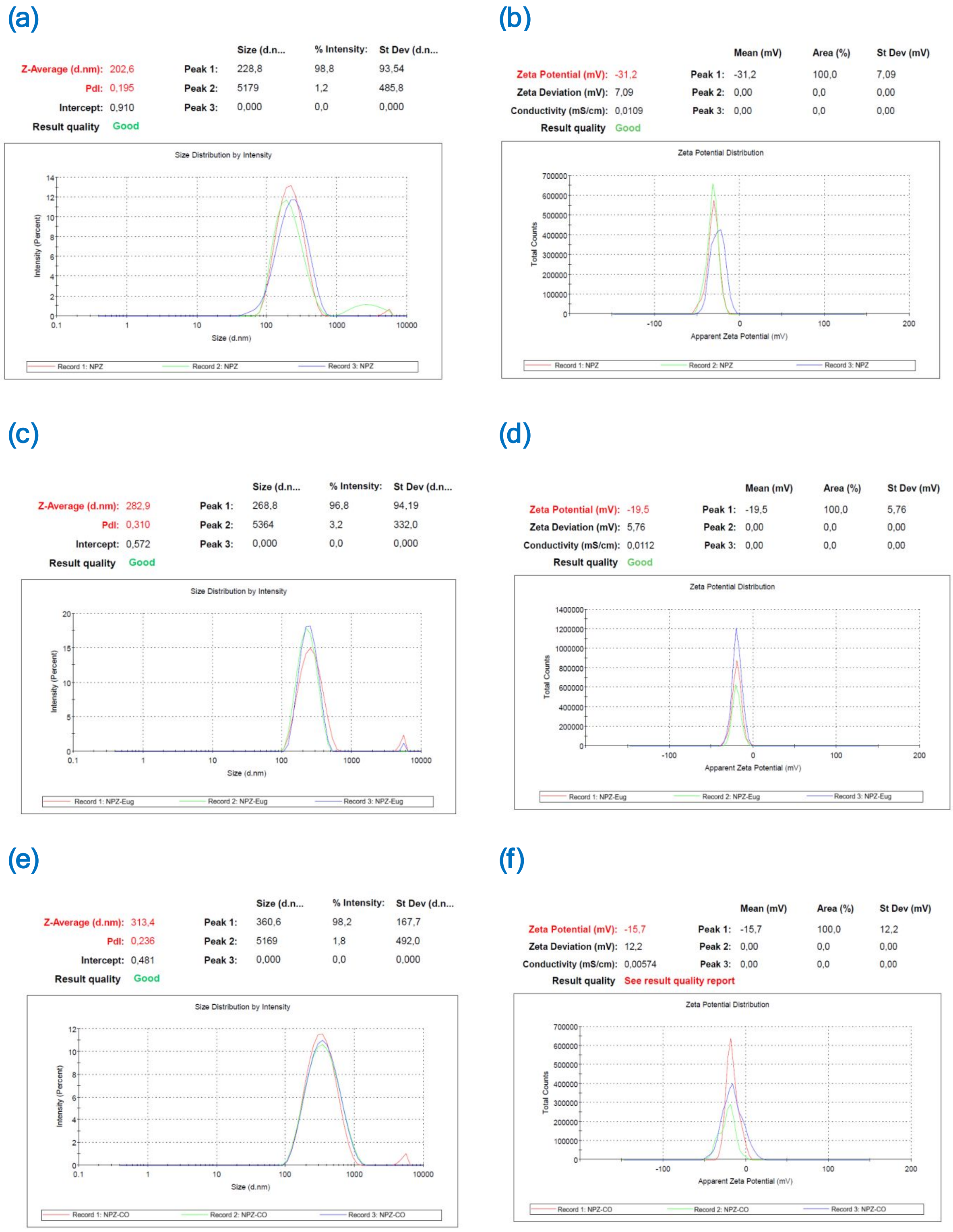

Figure S4 – Encapsulation efficiency (%EE) of nanoparticles samples stored at room temperature (RT) and under refrigeration (RF): Zein nanoparticles loaded with eugenol (NPZ-Eug); Zein nanoparticles loaded with clove essential oil (NPZ-CO); Zein nanoparticles (NPZ)

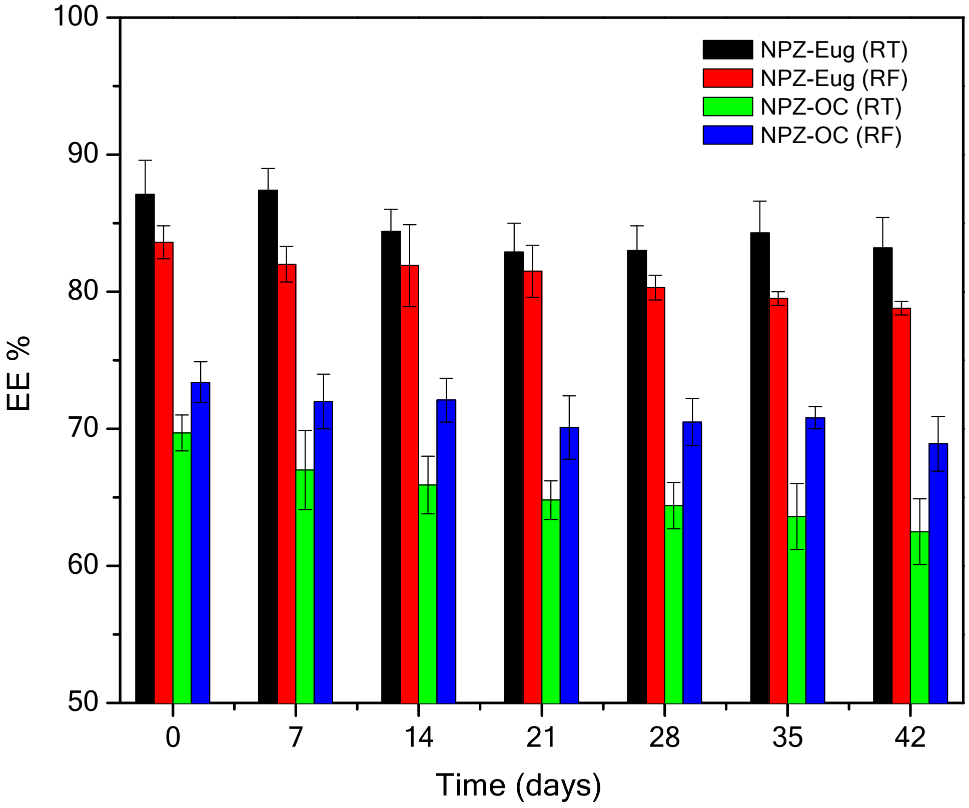

Figure S5 – Evaluation of pH and turbidity of nanoparticles over 42 days in accelerated stability tests: (a) Zein nanoparticles (NPZ); (b) zein nanoparticles loaded with eugenol (NPZ-Eug); (c) zein nanoparticles loaded with clove essential oil (NPZ-CO)

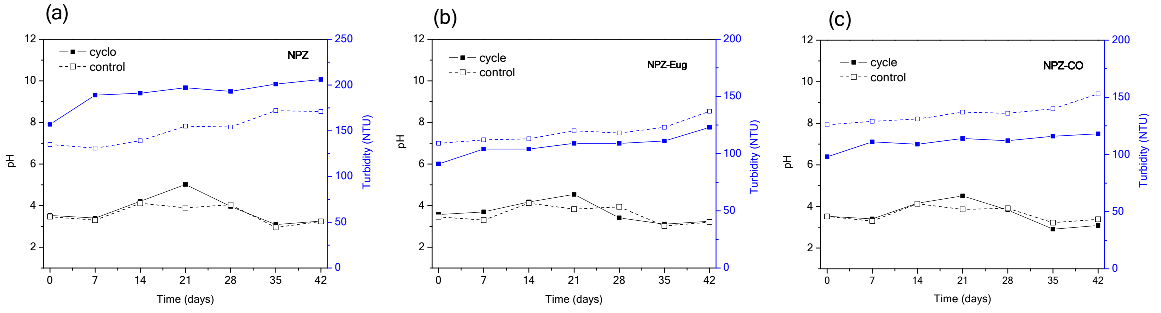

Figure S6 – Cumulative fraction of eugenol released from nanoparticles in PBS buffer (pH 7.4) over time, fitted to the kinetic models that best described the data (Korsmeyer–Peppas and Weibull): (a) NPZ-Eug; (b) NPZ-CO

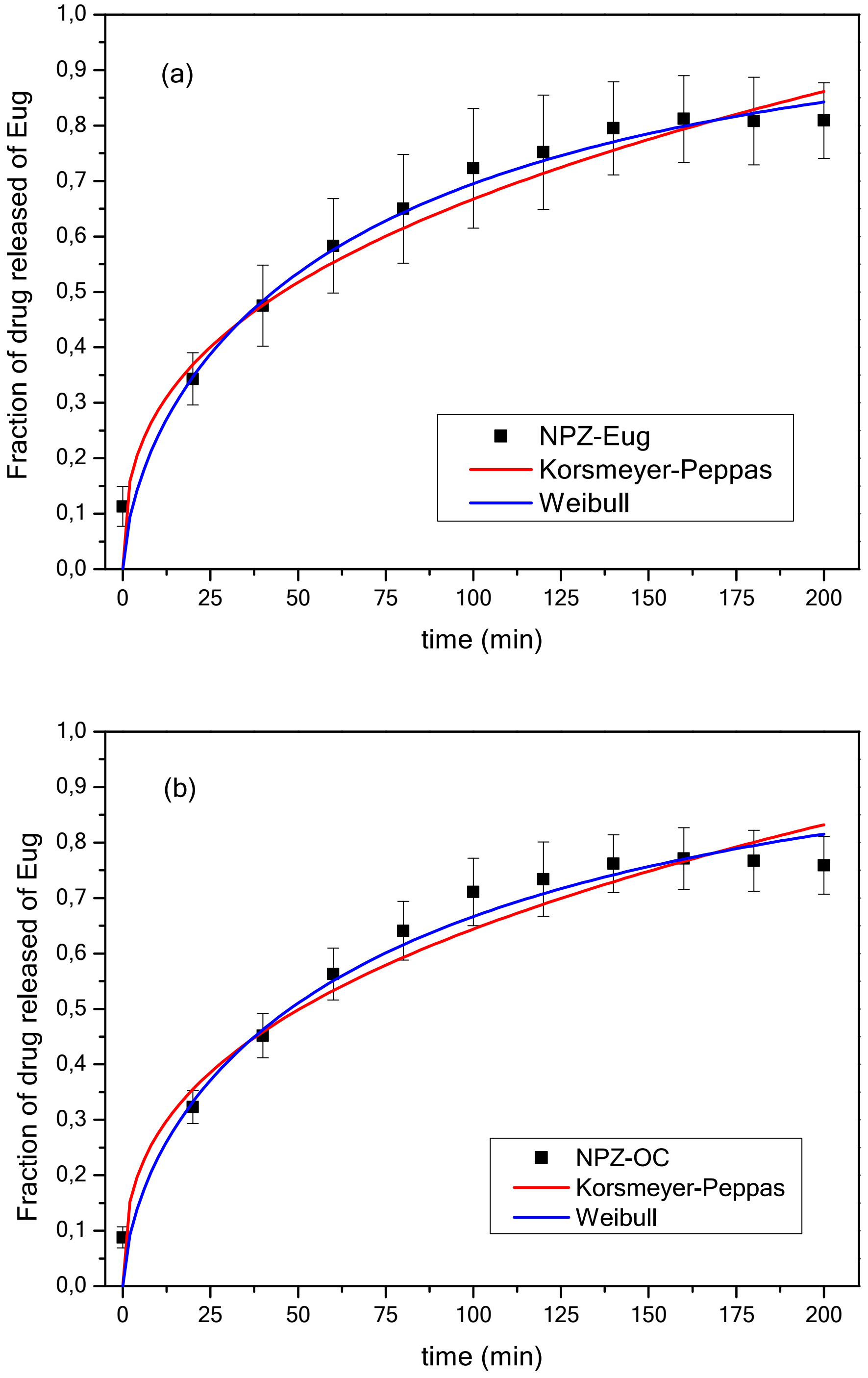

Figure S7 – Average percentage mortality of *Sitophilus zeamais* L. over 49 days of storage in maize grains treated with different samples: Zein nanoparticles loaded with eugenol (NPZ-Eug); Zein nanoparticles loaded with clove essential oil (NPZ-CO); Zein nanoparticles (NPZ); Clove essential oil (CO); Eugenol (Eug)

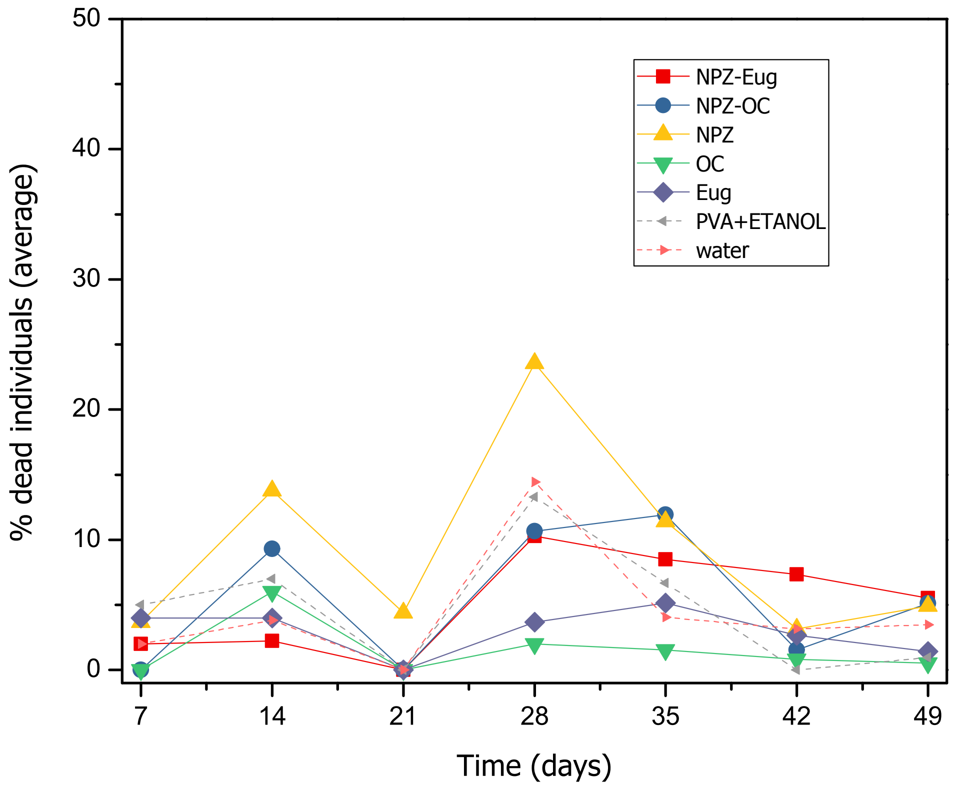

Figure S8 – Maize grains after 63 days: (a) healthy grains; (b) perforated grains; (c) grains with galleries

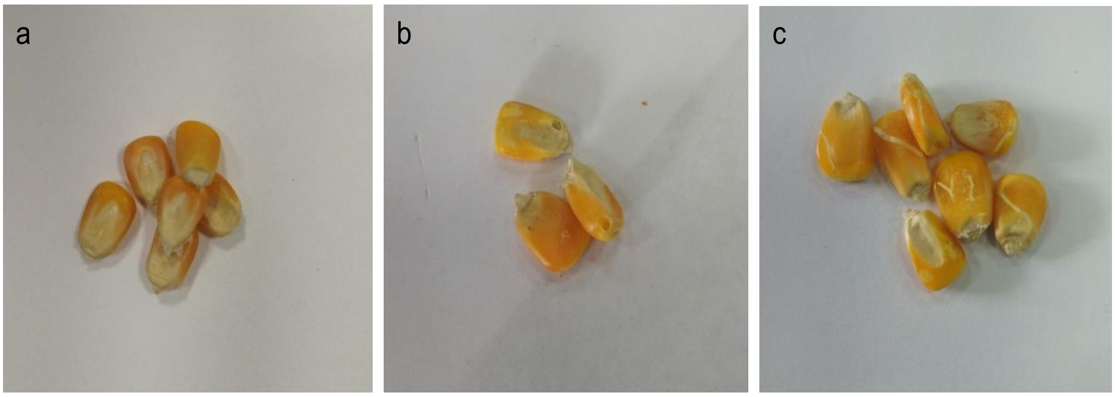

Table S1 – Summary of the statistical analyses applied to the different parameters evaluated in each assay

Parameter	Type of Analysis	Test Applied	Significance Level
**Mortality (direct and indirect contact)**	Nonparametric	Kruskal–Wallis + Dunn	1%
**Population development**	Nonparametric	Kruskal–Wallis + Dunn	1%
**Damaged grains**	Parametric	ANOVA (F-test) + Scott-Knott	5%
**Grain moisture content**	Parametric	ANOVA (F-test) + Scott-Knott	5%
**Repellency test**	Parametric	ANOVA (F-test) + Tukey	5%

Table S2 - Chemical compounds identified in clove essential oil

Compound	M.W. (g/mol)	GC-FID	Index Kovats		UV-Vis
		%Área	Literature	Experimental	Content (%)
**Eugenol**	164	79.32	1356	1351	78.60
**β-caryophyllene**	204	0.92	1417	1424	-
**Eugenyl acetate**	206	19.57	1524	1522	-
**β-caryophyllene oxide**	220	0.17	1582	1581	-

M.W. – Molecular weight; GC-FID – Gas chromatography coupled with flame ionization detector.

Table S3 – Comparative Summary of Release Profiles and Kinetic Parameters for Polymeric Delivery Systems

**Experimental Parameter**	**de Oliveira (2019)**	**Gomes (2011)**	**Lin (2024)**	**Seldlarikova (2024)**	**da Rosa (2015)**
Polymer matrix	Zein (data for NPGRL+EGL)	Poly(lactide-co-glycolide) (data for PLGA-E)	Rice Protein<br>(data for ERP7)	Poloxamer Micelles<br>(data for P184)	Zein<br>(data for NPT-T)
Surfactant / Stabilizer	Pluronic F68	PVA	n.a.	n.a.	Pluronic F68
Active compound	Eugenol + Geraniol	Eugenol	Eugenol	Eugenol	Thymol (T) or Carvacrol (C)
Temperature;<br>medium;<br>pH	25 ºC;<br>3% Pluronic F68;<br>n.a.	37 °C;<br>PBS (0.15 M, pH 7.4)	25 °C;<br>ultrapure water;<br>pH 7.0	25°C;<br>phosphate/Tween 80;<br>pH 7.5	n.a.<br>McIlvaine;<br>pH 7.4
Quantification technique	HPLC	HPLC-RP	n.a.	UV-Vis	HPLC
Burst release<br>(%, time)	n.a.	45%, ~ 5 h	~60%, ~ 2 h (⁑)	~ 18%, < 6 h	no
Sustained release<br>(%, time)	~50%, ~250 min (⁑)	~50%, ~ 12 h	~90%, ~ 3h	~ 30%, ~72 h	(C) ~ 48%, 8 h (⁑)<br>(T) ~ 55%, 8 h (⁑)
Saturation phase	~55-68%,<br>250-1500 min (⁑)	~50-60%,<br>~12-72 h	~90-92%,<br>~3-10 h	~ 30-50%,<br>~ 72-192 h	(C) ~ 48-50%, ~8-72h (⁑)<br>(T) ~ 55-60%, ~8-72h (⁑)
Mathematical model(s) tested	Zero-order,<br>First-order,	Second-order	First-order	First-order,<br>Higuchi,<br>Korsmeyer–Peppas	Korsmeyer–Peppas

	Higuchi, Korsmeyer–Peppas				
Best fit model (R^2)	Korsmeyer–Peppas (0.68)	Second-order (0.97)	First-order (n.a.)	Korsmeyer-Peppas (0.98)	Korsmeyer–Peppas (T = 0.98; C = 0.94)
Kinetic parameters best fit	$k = 1.695\ h^{-1}$<br>$n = 0.261$	$k_1 = 4.10\times10^{-4}\ s^{-1}$<br>$k_2 = 1.65 \times 10^{-6}\ s^{-1}$	$k = 0.01432\ min^{-1}$	$K_H = 0.0834\ h^{-1}$<br>$n = 0.420$	(T) $k = 0.287\ h^{-1}$;<br>$n = 0.635$<br>(C) $k = 0.140\ h^{-1}$,<br>$n = 1.144$
Release mechanism	Diffusion	Diffusion	Diffusion	Fickian diffusion	(T) anomalous Diffusion;<br>(C) = Diffusion

(n.a.) data not available; (⁑) data obtained from kinetic curve

Table S4 – Median number of *Sitophilus zeamais* individuals dead after 24, 48, and 72 hours following direct and indirect application of samples

Samples	Direct Application			Indirect Application		
	24h	48h	72h	24h	48h	72h
**NPZ-Eug**	0^{a}	1^{a}	1^{a}	1^{a}	1^{a}	1^{a}
**NPZ-CO**	0^{a}	0^{a}	0^{a}	0^{a}	0^{a}	0^{a}
**NPZ**	0^{a}	0^{a}	0^{a}	0^{a}	0^{a}	0^{a}
**Eug**	0^{a}	0^{a}	0^{a}	0^{a}	0^{a}	0^{a}
**CO**	0^{a}	0^{a}	0^{a}	0^{a}	0^{a}	0^{a}
**PVA+EtOH**	0^{a}	0^{a}	0^{a}	0^{a}	0^{a}	0^{a}
**Water**	0^{a}	0^{a}	0^{a}	0^{a}	0^{a}	0^{a}
**Treatments and controls**	0^{B}	0^{B}	0^{B}	0^{B}	0^{B}	0^{B}
**Insecticide**	8^{A}	10^{A}	10^{A}	1^{A}	6^{A}	6^{A}

Medians followed by the same lowercase or capital letter, separately, within columns do not differ significantly according to the Kruskal–Wallis and Dunn's tests, respectively, at the 1% significance level. Samples: Zein nanoparticles loaded with eugenol (NPZ-Eug); Zein nanoparticles loaded with clove essential oil (NPZ-CO); Zein nanoparticles (NPZ); Eugenol (Eug); Clove essential oil (CO); polyvinyl alcohol + ethanol (PVA+EtOH)

Table S5 – Median number of *Sitophilus zeamais* individuals at 3, 6, 12, 24, 48, 72 hours after application of different chemical compounds in choice arenas.

Arenas	Samples	3h	6h	12h	24h	48h	72h
**NPZ-Eug**	**NPZ-Eug**	3.7^{a}±2.91	3.1^{a}±2.48	4.1^{a}±3.58	2.3^{a}±2.14	4.4^{b}±2.23	5.3^{b}±2.21
	**CA**	14.6^{b}±3.60	13.1^{b}±2.97	7.0^{a}±3.83	1.6^{a}±2.88	1.1^{a}±1.46	0.4^{a}±0.79
	**Control**	1.1^{a}±1.07	3.7^{a}±4.07	8.9^{a}±5.34	16.1^{b}±4.71	14.3^{c}±3.04	14.4^{c}±2.51
**NPZ-CO**	**NPZ-CO**	2.4^{a}±2.51	2.3^{a}±1.89	3.9^{a}±2.85	4.7^{a}±3.59	5.0^{b}±3.37	5.7^{b}±3.09
	**CA**	16.3^{b}±3.09	15.7^{b}±1.98	8.9^{a}±2.73	2.0^{a}±2.31	1.0^{a}±1.41	0.9^{a}±1.21
	**Control**	1.3^{a}±1.60	2.0^{a}±1.83	8.0^{a}±4.62	13.3^{b}±3.40	14.0^{c}±3.96	13.4^{c}±3.10
**NPZ**	**NPZ**	1.7^{a}±0.95	2.4^{a}±2.07	4.6^{a}±2.07	6.9^{ab}±4.18	7.7^{b}±3.30	6.9^{b}±3.72
	**CA**	16.9^{b}±1.77	14.6^{b}±2.82	9.6^{a}±4.43	2.0^{a}±2.31	0.6^{a}±0.79	0.3^{a}±0.49
	**Control**	1.4^{a}±1.40	3.3^{a}±1.80	6.6^{a}±3.82	11.1^{b}±4.56	12.4^{b}±3.60	12.9^{c}±3.89
**CO**	**CO**	7.0^{a}±4.47	7.1^{a}±5.30	7.1^{a}±6.62	8.7^{b}±6.07	8.9^{b}±5.37	7.7^{b}±5.47
	**CA**	9.3^{a}±5.15	8.7^{a}±5.74	8.6^{a}±5.68	1.0^{a}±1.15	0.3^{a}±0.49	0.4^{a}±0.53
	**Control**	3.7^{a}±3.35	4.1^{a}±3.34	5.1^{a}±3.53	10.3^{b}±5.47	10.9^{b}±5.64	12.0^{b}±5.42
**Eug**	**Eug**	1.9^{a}±2.04	2.9^{a}±2.04	8.7^{a}±5.47	14.1^{b}±6.34	9.7^{b}±4.31	8.3^{b}±3.77
	**CA**	17.7^{b}±2.75	16.0^{b}±3.06	5.1^{a}±2.48	0.6^{a}±0.53	0.3^{a}±0.49	0.6^{a}±0.79
	**Control**	0.4^{a}±0.79	1.1^{a}±1.86	6.1^{a}±6.34	5.3^{ab}±6.32	10.0^{b}±4.43	11.1^{b}±3.76
**PVA+ EtOH**	**PVA+EtOH**	4.0^{a}±3.21	3.6^{a}±4.47	4.1^{a}±4.06	7.4^{ab}±3.60	7.6^{b}±3.74	7.6^{b}±3.60
	**CA**	11.4^{a}±5.53	9.7^{a}±4.50	8.7^{a}±4.54	1.4^{a}±1.99	0.6^{a}±1.13	0.9^{a}±1.46
	**Control**	4.6^{a}±4.04	6.6^{a}±3.87	7.3^{a}±4.57	11.1^{b}±4.63	11.9^{b}±4.26	11.6^{b}±4.58

Mean ± SD followed by the same letter within each evaluation period do not differ significantly by Tukey's test at the 5% significance level. Samples: Zein nanoparticles loaded with eugenol (NPZ-Eug); Zein nanoparticles loaded with clove essential oil (NPZ-CO); Zein nanoparticles (NPZ); Eugenol (Eug); Clove essential oil (CO); polyvinyl alcohol + ethanol (PVA+EtOH); Center of the Arena (CA).

Table S6 – Summary of the analysis of variance of *Sitophilus zeamais* L. preference data at 3, 6, 12, 24, 48, and 72 hours after application of different chemical compounds in choice arenas.

Sources of Variation	DF	3h	6h	12h	24h	48h	72h
				**NPZ-Eug**			
**Blocks**	6	0.048	0.023	0.038	0.097	0.037	0.024
**Treatments**	2	11.680**	7.279**	1.448^{ns}	14.678**	11.168**	13.276**
**Residual**	12	0.346	0.684	0.898	0.587	0.280	0.186
				**NPZ-CO**			
**Blocks**	6	0.081	0.031	0.024	0.025	0.048	0.029
**Treatments**	2	15.353**	13.128**	1.853^{ns}	8.135**	11.008**	10.697**
**Residual**	12	0.385	0.260	0.549	0.515	0.461	0.336
				**NPZ**			
**Blocks**	6	0.057	0.110	0.032	0.079	0.104	0.059
**Treatments**	2	16.585**	9.822**	1.313^{ns}	5.864**	10.673**	11.781**
**Residual**	12	0.158	0.309	0.517	0.707	0.347	0.470
				**CO**			
**Blocks**	6	0.122	0.117	0.130	0.082	0.023	0.025
**Treatments**	2	2.187^{ns}	1.327^{ns}	0.699^{ns}	7.079*	10.047**	10.144**
**Residual**	12	1.025	1.175	1.350	1.089	0.862	0.778
				**Eug**			
**Blocks**	6	0.080	0.088	0.019	0.047	0.011	0.012
**Treatments**	2	20.361**	14.778**	0.583^{ns}	11.603**	10.423**	9.651**
**Residual**	12	0.218	0.337	1.401	1.227	0.498	0.391
				**PVA + EtOH**			
**Blocks**	6	0.053	0.014	0.017	0.106	0.078	0.089
**Treatments**	2	3.739^{ns}	2.806^{ns}	1.564^{ns}	7.154**	10.082**	8.990**
**Residual**	12	0.990	1.127	1.084	0.677	0.556	0.624

DF = degrees of freedom. Data were square root transformed as $\sqrt{(x + 1)}$. *5% significance level; **1% significance level; ns = not significant. The numerical values reported correspond to mean squares. Samples: Zein nanoparticles loaded with eugenol (NPZ-Eug); Zein nanoparticles loaded with clove essential oil (NPZ-CO); Zein nanoparticles (NPZ); Eugenol (Eug); Clove essential oil (CO); polyvinyl alcohol + ethanol (PVA+EtOH)

Table S7 - Medians of *Sitophilus zeamais* L. mortality percentage during the storage period (up to 49 days) of maize grains treated with different samples

Samples	7 days	14 days	21 days	28 days	35 days	42 days	49 days
NPZ-Eug	0^a	0^a	0^a	10.0^a	7.7^a	0.9^a	3.3^a
NPZ-CO	0^a	8.3^a	0^a	0^a	4.8^a	1.2^a	1.6^a
NPZ	0^a	11.1^a	0^a	27.3^a	15.4^a	2.5^a	2.9^a
Eug	0^a	0^a	0^a	0^a	0^a	2.1^a	1.2^a
CO	0^a	0^a	0^a	0^a	0^a	0^a	0.8^a
PVA+EtOH	0^a	0^a	0^a	9.1^a	0^a	0^a	0^a
Water	0^a	0^a	0^a	20.0^a	5.3^a	3.3^a	2.1^a

Medians followed by the same lowercase letter within columns do not differ significantly according to the Kruskal–Wallis test at the 5% significance level. Samples: Zein nanoparticles loaded with eugenol (NPZ-Eug); Zein nanoparticles loaded with clove essential oil (NPZ-CO); Zein nanoparticles (NPZ); Eugenol (Eug); Clove essential oil (CO); polyvinyl alcohol + ethanol (PVA+EtOH)

Tabela S8 - Summary of the analysis of variance of damaged and undamaged maize grains caused by *Sitophilus zeamais* L., and grain moisture content after 63 days of storage following treatment with different samples.

Sources of Variation	DF	Damaged grains (g)	Undamaged grains (g)	Moisture content (%)
**Treatments**	7	2008.721**	2507.729**	0.907^{ns}
**Residual**	32	375.154	526.135	0.641

*5% significance level; **1% significance level; ns = not significant. The numerical values reported correspond to mean squares. The numerical values reported correspond to mean squares.

References

(1) de Oliveira, J.L. et al. Association of zein nanoparticles with botanical compounds for effective pest control systems. Pest Manag Sci 2019; 75: 1855–1865

(2) Gomes, C.; Moreira, R. G.; Castell- Perez, E. Poly (DL-lactide-co-glycolide)(PLGA) nanoparticles with entrapped trans-cinnamaldehyde and eugenol for antimicrobial delivery applications. Journal of Food Science, 2011; 76: N16-N24.

(3) Lin, C. et al. Salting-out effect-mediated size-control of protein nanoparticles towards controllable microstructures for sustained release of eugenol. Food Chemistry, 2024; 439: 138080.

(4) Seldlarikova, J. et al. Poloxamer-Based Mixed Micelles Loaded with Thymol or Eugenol for Topical Applications. ACS Omega 2024; 9: 23209−23219.

(5) da Rosa, C. G. et al. Characterization and evaluation of physicochemical and antimicrobial properties of zein nanoparticles loaded with phenolics monoterpenes. Colloids and Surfaces A: Physicochemical and Engineering Aspects, 2015; 481: 337-344.
